# Short linear motifs—underexplored players driving *Toxoplasma gondii* infection

**DOI:** 10.1128/spectrum.00724-26

**Published:** 2026-08-05

**Authors:** Jesús Alvarado Valverde, Karine Lapouge, Arne Boergel, Kim Remans, Katja Luck, Toby J. Gibson

**Affiliations:** 1 Structural and Computational Biology Unit, European Molecular Biology Laboratory 9471, Heidelberg, Germany; 2 Faculty of Biosciences, Collaboration for a joint PhD degree between EMBL and Heidelberg University 98938, Heidelberg, Germany; 3 Institute of Molecular Biology (IMB) gGmbH 378044, Mainz, Germany; 4 Protein Expression and Purification Core Facility, European Molecular Biology Laboratory 9471, Heidelberg, Germany; Johns Hopkins School of Public Health, Baltimore, Maryland, USA; Johns Hopkins University, Baltimore, Maryland, USA

**Keywords:** protein-protein interactions, *Toxoplasma gondii*, short linear motifs, host-pathogen interactions

## Abstract

Pathogens infect hosts by interacting with host proteins and exploiting their functions to their advantage. Short linear motifs, small functional regions within intrinsically disordered protein regions, are common mediators of host-pathogen protein interactions. While motifs have been more extensively studied in viruses and bacteria, the extent to which eukaryotic unicellular parasites use motifs during infection remains poorly explored. *Toxoplasma gondii* is a widespread intracellular apicomplexan parasite capable of infecting all warm-blooded animals and invading any of their nucleated cells. *Toxoplasma*’s secreted proteins are key in interacting with host proteins during infection, making them potential sources for motifs. To study the role of motifs in *Toxoplasma gondii* infection, we curated 19 known motif instances in *Toxoplasma* proteins from the scientific literature. To identify more motifs in *Toxoplasma-*secreted proteins, we developed a computational pipeline that predicts and annotates putative motif matches with structural and functional features. Using this approach, we identified 24,097 motif matches within 295 proteins from secretory organelles. We highlight strategies to further prioritize likely functional motif matches by focusing on integrin motifs, degrons, TRAF6-binding motifs, and 42 confirmed secreted proteins. We subjected peptides containing four predicted TRAF6-binding motifs to experimental validation, supporting the predicted motifs in the *Toxoplasma* proteins RON10 and GRA15. Our motif predictions provide a valuable resource for generating hypotheses and designing experiments to study infection mechanisms. The characterization of motifs in *Toxoplasma* will be key to understanding the molecular principles underlying its broad host range and more comprehensive apicomplexan infection strategies.

**IMPORTANCE:**

*Toxoplasma gondii* is a widely distributed intracellular parasite that achieves successful infection by interacting with different host cell proteins. Short linear motifs are small functional modules found in unstructured protein regions and recognized by folded protein domains. Given that unstructured protein regions are a common feature of *Toxoplasma*’s proteins, we hypothesize that motifs play important roles during its infection cycle. Here, we highlight the role of motifs during the *Toxoplasma* host cell invasion cycle through a curated set of motif examples. Through a computational pipeline, we predict thousands of motifs in secreted proteins, outline strategies for working with these predictions, and finally experimentally test peptides containing a motif involved in the innate immune response, successfully supporting the potential binding of two motifs. Our work provides a resource for further motif testing in *Toxoplasma* proteins, aiming at understanding the molecular mechanisms of the organism’s infection strategies and its broad host range.

## INTRODUCTION

Intracellular pathogens, such as viruses, bacteria, or eukaryotic parasites, interact with host cell components throughout their infection cycles. These pathogens can attach to the host membrane and, when internalized, they can interact with the host cytoskeleton and organelles, as well as with its export and translation machinery (1–3). By interfering with host biological processes, pathogens modulate the host immune response, replicate, and disperse. Pathogen proteins play a central role in these interactions. A common mode of protein binding that mediates host-pathogen protein-protein interactions (PPIs) consists of a folded domain, e.g., in host proteins, and short linear motifs (hereafter referred to as motifs) in pathogen proteins (2–4). These motifs are usually between 3 and 10 amino acids long and occur in intrinsically disordered regions of proteins (IDRs) (5, 6). Motifs mediate protein interactions that primarily participate in regulatory and cell signaling processes. Motifs define sites for post-translational modifications, subcellular protein localization, as well as substrate recognition signals for enzymes such as E3 ligases, proteases, phosphatases, etc. (5). Motifs are unstructured in their unbound state and often adopt an alpha-helix or beta-strand upon binding to partner proteins (6). Because only a few mutations are needed to create a motif, they can evolve in much shorter evolutionary timescales compared with folded protein regions (7). Thus, pathogens have evolved to exploit these host motif systems to infect their hosts, probably because motifs are short and easily emerge in proteins (7) but also because of their many functional roles in cell signaling. Many instances of host motif mimicry have been characterized for viruses and bacteria (2, 3, 8–11). For example, a strain of the gastroenteritis-causing Gram-negative bacteria *Vibrio cholerae* secretes the effector protein VopF, which contains actin-interacting motifs, into host cells. These motifs allow VopF to mimic host actin-nucleating proteins, like SPIRE, to interact with actin and promote their nucleation, leading to cell surface protrusions, which in turn would promote an efficient intestinal colonization (12). Another example comes from the herpesvirus tegument protein UL37, which leads to the activation of the NF-kB immune response early during infection via a TRAF6-binding motif. The activation of NF-kB would then be co-opted for the transcription of other viral genes (13). Although there have been some studies focusing on intracellular eukaryotic parasites (14, 15), the extent to which parasites use motifs is less understood. *Toxoplasma gondii,* a member of the Apicomplexa phylum of unicellular parasites, has one of the proteomes with higher levels of IDRs among eukaryotes (16, 17), making it a good candidate to discover and characterize the role of motifs in its proteins during parasitic infection.

*Toxoplasma* is a successful eukaryotic unicellular parasite, as it can infect any warm-blooded animal and proliferate within any of its nucleated cells. It is estimated that a third of the human population has been infected with Toxoplasma (18). Even though most of the infections in humans are asymptomatic, immunocompromised individuals and susceptible populations can develop different forms of toxoplasmosis, from influenza-like symptoms to severe phenotypes, such as encephalitis, making *Toxoplasma* a global health burden (18, 19). *Toxoplasma*’s definitive hosts are cats and all members of the feline family, where it reproduces sexually in their gut to then disseminate in the environment. In all other hosts, such as all mammals and birds, *Toxoplasma* reproduces asexually in different cell types to multiply its numbers and disseminate along the food chain (20). Having multiple hosts means that *Toxoplasma* has the molecular tools not only to infect different host cells but also to deal with diverse cell programs and immune responses to thrive in each of its hosts. Motifs in *Toxoplasma* proteins might play important roles in mediating the adaptation to the many different host environments this parasite encounters.

**Fig 1:**
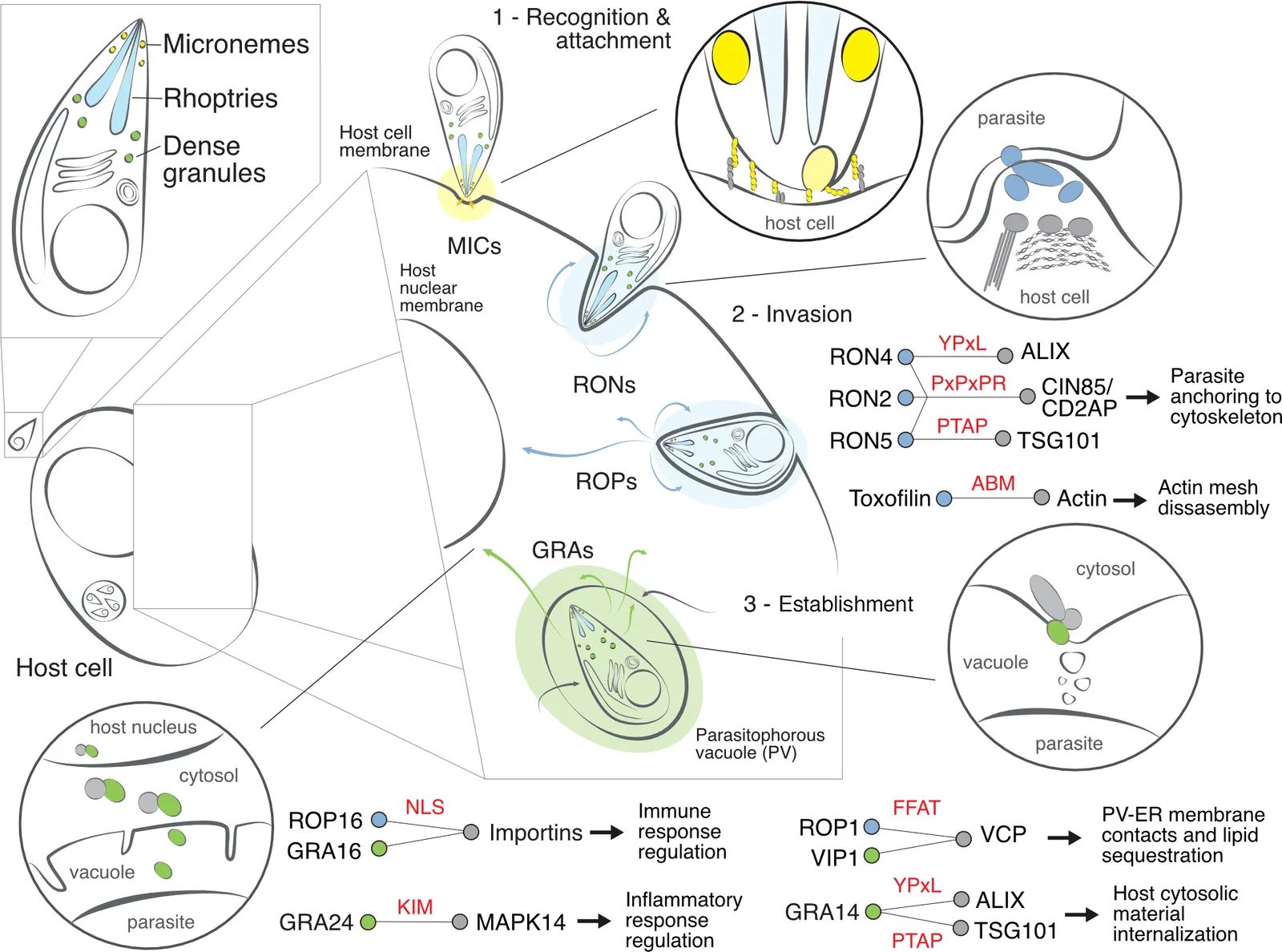
Motif use and hijacking during the *Toxoplasma* infection process. *Toxoplasma gondii* secretory organelles and infection cycle illustrating different host-pathogen interaction contexts, known motif instances, and their infection outcomes. Motif names are highlighted in red, proteins from micronemes are highlighted in yellow, those from rhoptries in blue, and those from dense granules in green.

Once consumed by its host, *T. gondii* cell invasion cycles depend on the sequential secretion of proteins contained in its specialized infection organelles: the micronemes, rhoptries, and dense granules (Fig. 1A). Microneme proteins, or MICs, are targeted to the parasite cell surface and are involved in host recognition and attachment, while rhoptry proteins, termed RONs and ROPs, are secreted into the host cytoplasm and are involved in host invasion and niche establishment (Fig. 1B). During the invasion process, a parasitophorous vacuole (PV) is created from the invagination of the host cell membrane (21). From within this space, *Toxoplasma* creates a suitable environment and continues to secrete proteins from secretory vesicles called dense granules (22). These proteins, mostly known as GRAs, are secreted either into the PV lumen, inserted into its membrane (PVM), or are exported into the host cytoplasm and sometimes targeted to the host nucleus, helping the parasite in modulating the host cell’s regulatory processes to proliferate (23). A subset of these proteins is secreted into the host cytoplasm and can interact with host proteins throughout the infection cycle, including host cell receptors, cytoskeletal proteins and transcription factors (24, 25). These host-parasite protein interactions are the subject of continuous study, serve as a reference to study other apicomplexans, and are promising targets for the development of therapies. Knowledge about motifs in *Toxoplasma*-secreted proteins would increase our mechanistic understanding of *Toxoplasma* infection strategies.

To identify motifs in *Toxoplasma gondii* proteins, we collected motif instances in *T. gondii* from the literature, predicted occurrences of known motif types in proteins from its secretion organelles through the development of an integrative discovery pipeline, and tested the binding of peptides containing predicted TRAF6-binding motifs *in vitro,* which might have relevance in the regulation of the innate immune response. The available motif predictions along with their annotations can guide further functional studies in *Toxoplasma* and other apicomplexan parasites to understand mechanisms of infection and provide further examples of motif repurposing among intracellular pathogens.

## RESULTS

### Curation of known motifs in *Toxoplasma gondii*secreted proteins

We collected 19 experimentally validated motif instances from 10 *Toxoplasma* proteins that interact with host proteins from the scientific literature that serve as a reference of the current motif knowledge in *Toxoplasma gondii* cell invasion (Table 1, Fig. 1). The Eukaryotic Linear Motif (ELM) resource (http://elm.eu.org/) is a reference point for curated eukaryotic motifs from the scientific literature. Based on motif sequence patterns, structures, binder information, and functional annotations, curators define motif types and group motif types into broader motif classes within ELM (26). Out of the 19 curated *Toxoplasma* motifs, two kinase-interacting motifs (KIMs) were already annotated as substrate recognition sites in ELM (referred to as docking (DOC) motif classes, Table 1). Eleven motif instances were novel with respect to motif instances annotated in ELM and correspond to motif scaffolding classes in ELM (referred to as LIG classes, which mediate the formation of signaling protein complexes). The remaining six novel motif instances are similar but do not match the defined sequence patterns (regular expressions) of motif types in ELM (Table 1). These motifs are similar to one scaffolding class (LIG) and to two subcellular localization classes (referred to as TRG in ELM). We did not annotate motif instances in *Toxoplasma*-secreted proteins reported as motifs of cleavage sites (CLV in ELM), degradation signals (DEG in ELM), or sites for post-translational modifications (MOD in ELM). As detailed below, these curated 19 motif instances function throughout parasite host cell invasion and establishment (Fig. 1, Table 1).

**TABLE 1:** Curated motif instances from the literature with experimental support^*a*^

Protein name	ToxoDB (UniProt)	Motif name (ELM ID)	Host protein (domain name)	Instances (motif ranges)	Annotation	Source
Rhoptry proteins						
RON2	TGME49_300100 (B6KV60)	PxPxPR (LIG_SH3_CIN85_PxpxPR_1)	CIN85 or CD2AP (SH3)	1 (59–64)	Novel ELM instance	(27)
RON4	TGME49_229010 (S8F0B5)	PxPxPR (LIG_SH3_CIN85_PxpxPR_1)	CIN85 or CD2AP (SH3)	3 (115–120, 131–136, 260–265)	Novel ELM instances	(27)
		LYPxL (LIG_LYPXL_S_1)	Alix (V-shaped)	2 (35–39, 170–174)	Novel ELM instances	(27)
RON5	TGME49_311470 (S8ERS3)	PxPxPR (LIG_SH3_CIN85_PxpxPR_1)	CIN85 or CD2AP (SH3)	1 (96–101)	Novel ELM instance	(27)
		PTAP (LIG_PTAP_UEV_1)	Tsg101 (UEV)	2 (586–591, 620–626)	Novel ELM instances	(27)
ROP1	TGME49_309590 (A0A125YP48)	FFAT (TRG_ER_FFAT_1 like)	VAP (MSP)	1 (163–172)	Not in ELM	(28)
ROP16	TGME49_262730 (Q3YJR5)	NLS (TRG_NLS_MonoExtN_4)	Exportin (Arm)	1 (339–344)	Not in ELM	(29)
Toxofilin	TGME49_214080 (S8G4J8)	Actin-binding motif (LIG_Actin_WH2_1 like)	Actin	1 (103–161)	Not in ELM	(30)
Dense granule proteins						
GRA14	TGME49_239740 (B5TVE8)	LYPxL (LIG_LYPXL_S_1)	Alix (V-shaped)	1 (394–398)	Novel ELM instance	(31)
		PTAP (LIG_PTAP_UEV_1)	Tsg101 (UEV)	1 (383–388)	Novel ELM instance	(31)
GRA16	TGME49_208830 (Q1JSN3)	NLS (TRG_NLS_MonoExtN_4)	Exportin (Arm)	2 (69–76, 250–256)	Not in ELM	(32)
GRA24	TGME49_230180 (A0A125YG37)	KIM (DOC_MAPK_GRA24_9)	MAPK (Kinase)	2 (441–455, 520–534)	Previously annotated	(33)
VIP1	TgME49_233220 (S8F2E2)	FFAT (TRG_ER_FFAT_1 like)	VAP (MSP)	1 (529–534)	Not in ELM	(34)

^*a*^ *Toxoplasma* motif instances are provided with interacting host proteins and domains. Protein IDs correspond to the ToxoDB and UniProt accessions, while motif IDs correspond to ELM motif types. DOC (docking), LIG (ligand, scaffolding site), TRG (targeting, subcellular localization). Motif names: KIM (kinase-interacting motif) and NLS (nuclear localization signal).

Rhoptry proteins RON2, RON4, and RON5 each contain PxPxPR motifs. During host cell invasion, these motifs help the parasite connect its tight junction (TJ) complex to the host cytoskeleton (27) (Fig. 1). The PxPxPR motifs bind to the SH3 domain of host adaptor proteins CIN85 or CD2AP, which are involved in cytoskeletal rearrangements, vesicle trafficking, and endocytosis (35, 36). Additionally, RON4 and RON5 contain instances of the motif types LYPxL and PTAP. The LYPxL motif mediates the interaction of proteins with ALIX, while the PTAP motif binds to the UEV domain of class E vacuolar sorting protein TSG101. ALIX and TSG101 are both key components of the ESCRT membrane remodeling system, and during *Toxoplasma* cell invasion, they are also recruited to the TJ for efficient cell entry (27, 37). This process is also aided by parasite proteins, such as Toxofilin, which binds to host actin through an actin-binding motif (ABM) that is similar in sequence pattern to the annotated WH2-binding motif in ELM (30). Toxofilin sequesters actin and promotes the disassembly of the host cortical actin meshwork, facilitating efficient internalization (38).

Once *Toxoplasma* is established within the host cell, ROP1 and dense granule proteins, VIP1 and GRA14, are secreted and exported to the parasitophorous vacuole membrane (PVM) (Fig. 1). ROP1 and VIP1 both contain FFAT motifs (similar in sequence motif pattern to the FFAT motif type in ELM) that modulate the targeting of proteins to the endoplasmic reticulum via its interaction with vesicle-associated membrane protein (VAMP)-associated proteins (VAPs). As ROP1 and VIP1 are inserted in the PVM, their interaction with VAPs leads to the association of the PVM with the host ER membrane. *Toxoplasma* exploits this proximity to sequester the lipids necessary for its vacuole and plasma membrane biogenesis (28, 34). GRA14 harbors both LYPxL and PTAP motifs in its C-terminal region. Since GRA14 is also inserted into the PVM with its C-terminus facing the host cytosol, the motifs can recruit ALIX and TSG101, which, in this case and differently from TJ proteins, help *Toxoplasma* sequester host cytosolic material through the budding of its PVM (31) or by the internalization of large cargos, such as host Rab vesicles, into the PV (39).

Further, during colonization and formation of an intracellular niche, more parasite proteins are secreted into the host cytosol by the parasite machinery and subsequently may be transported to the host nucleus by the host cellular machinery. Within the host cytosol, secreted proteins may interact with host proteins via motifs. The intrinsically disordered protein GRA24 contains two KIM instances that bind to mitogen-activated protein kinase p38α (MAPK14). These two motifs allow GRA24 to bind to a MAPK14 dimer, forming a stable complex that leads to the expression of proinflammatory factors, which in turn modulate parasite replication (33, 40). The correct export of dense granule protein GRA16 to the cytosol, as well as of other GRA proteins, is dependent on the cleavage of a short linear motif termed TEXEL (Toxoplasma export element) by parasite aspartyl protease ASP5, in a similar way as the *Plasmodium* PEXEL export motif (32, 41) and analyzed in detail in (42). These TEXEL export motifs are examples of internal use of motifs by *Toxoplasma* for the maturation of secreted proteins and not of motif hijacking for interacting with host proteins (thus not included in our curated list). Once in the host cytosol, GRA16, as well as rhoptry protein kinase ROP16, are imported into the host nucleus via nuclear localization signals (NLS). ROP16 activates signal transducer and activator of transcription (STAT) signaling pathways to inhibit host cell death and modulate the production of interleukins (29, 43). Within the nucleus, GRA16 regulates genes involved in cell cycle progression (32). This set of curated motif instances exemplifies their roles during important stages of the *Toxoplasma* infection cycle. Nevertheless, the small number of known motifs as well as their occurrence in a few proteins, highlight the need and potential to find and characterize many more motif instances to facilitate further mechanistic studies of *Toxoplasma* pathogenicity.

### Prediction of motif instances in *Toxoplasma*-secreted proteins

**Fig 2:**
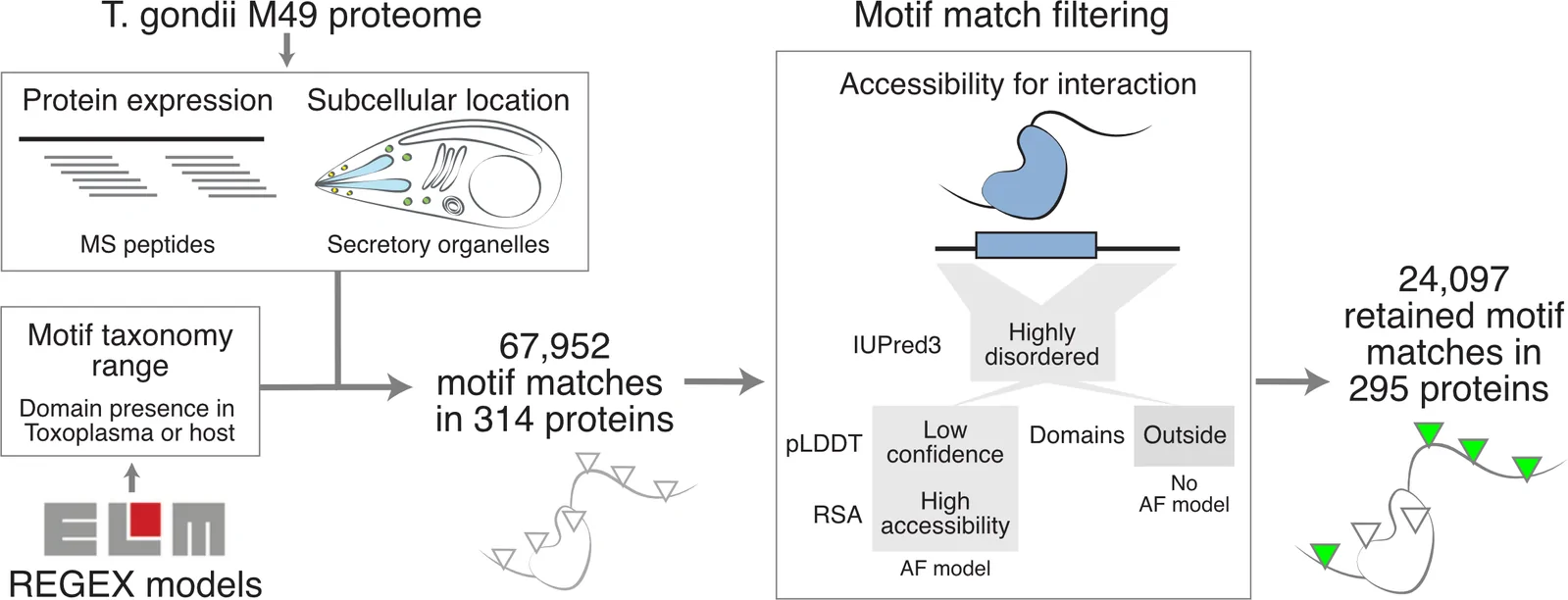
Motif prediction pipeline. Diagram outlining the protein and motif type selection process for motif prediction, as well as the filtering of motif matches via structural accessibility.

**Fig 3:**
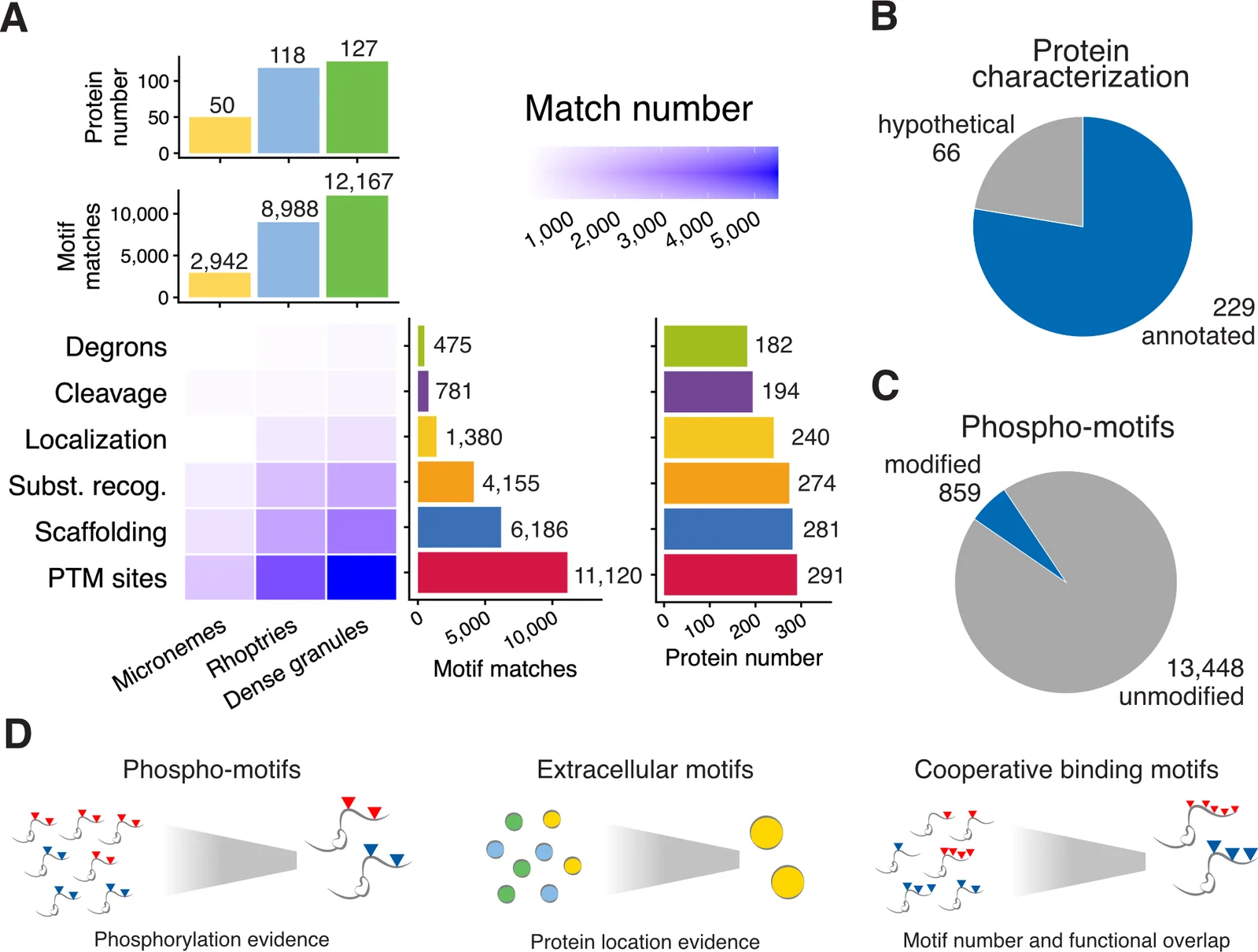
Summary of predicted motif matches. (**A**) Number of filtered motif matches from putatively secreted proteins across different motif categories. (**B**) Split of proteins with filtered matches according to their annotation. (**C**) Split of filtered phospho-motifs according to whether they have phosphorylation evidence. (**D**) Additional motif filtering strategies according to motif type functions and properties.

To identify novel motif instances that function in the *Toxoplasma* infection process via interaction with host proteins, we focused on *Toxoplasma gondii* proteins from the strain ME49 that can potentially be secreted into host cells. To this end, we aimed to identify proteins that reside in micronemes, rhoptries, and dense granules, and thus, have a higher probability of interacting with host proteins either in the extracellular (for micronemes) or intracellular space after secretion into the host cytosol or upon incorporation into membranes. To obtain information on the subcellular localization of Toxoplasma proteins, we used the hyperLOPIT data set, which was generated from mass spectrometric analysis of cellular fractionations corresponding to different *Toxoplasma*-relevant subcellular compartments (44). All proteins identified in hyperLOPIT to locate to secretory organelles or annotated as such were subsequently filtered for those with evidence of expression using mass spectrometry data sets from ToxoDB (45), ultimately defining a set of 314 *Toxoplasma* proteins. To predict motif instances in these proteins*,* we developed a computational pipeline based on sequence pattern searches using regular expressions as well as structural and functional annotations of predicted motifs (Fig. 2). Because motifs are short and often only a few instances for a given motif type are known, regular expressions are commonly used to describe motif sequence patterns and search for motif matches in protein sequences (46). To find putative motif instances, we used regular expressions defined for annotated motif classes in the ELM database (see Materials and Methods). We scanned the selected proteins with 297 regular expressions corresponding to motif classes with potential interaction with domains present either in *Toxoplasma* or in the host using the taxonomic range annotations provided by the ELM resource, i.e., excluding motif classes that would only interact with domains present in fungi or plants or that are known to be absent in Toxoplasma, such as the PEXEL motif as described earlier. This motif search resulted in a total of 67,952 motif matches in all 314 proteins. Since motifs occur primarily in IDRs and need to be accessible for binding to the partner proteins, we deployed structural annotations to further restrict ourselves to motif matches in IDRs that are more likely to correspond to functional motif matches. To this end, we used the software IUPRed3 (47), which predicts which protein regions are likely ordered or disordered, as done previously in other motif discovery studies (48, 49). To further evaluate the accessibility of predicted motif residues for interaction, we took protein structural models available in the EBI AlphaFold database (50). Using these structural models, we evaluated motif accessibility through low pLDDT values provided by AlphaFold, which indicate regions of disorder. We also used the structural models to calculate relative solvent accessibility (RSA) for the folded regions, through which motif matches in inaccessible regions were filtered out. In cases where a structural model was not available, we excluded motif matches that fell within annotated folded domain boundaries as recorded in UniProt (51) (Fig. 2). Using these filtering steps, we retained 24,097 motif matches within 295 proteins from secretion organelles (Fig. 2 and 3A) (Table S1).

Our computational pipeline captured 14 of the 19 curated motif instances within our retained motif matches. Two PTAP motifs in RON5 were missed because they had low IUPred scores indicative of ordered protein regions. The FFAT motif in ROP1 and the actin-binding motif in Toxofilin were not captured because their sequences did not exactly match any ELM regular expression. These last two examples highlight that current annotated motif types in the ELM resource do not fully capture the diversity of motifs in *Toxoplasma* proteins. Our curated motif instances could thus be used to reevaluate and extend ELM motif type annotations. Focusing on the *Toxoplasma* proteins with curated motif instances, we predicted a previously unreported PxPxPR motif in the N-terminal region of RON4, as well as two SxIP motifs. SxIP motifs modulate the binding to microtubule plus-end tracking proteins (+TIPs), which have an important role in chromosome segregation, cell signaling and organelle trafficking (52). SxIP motifs are already known to be exploited by the Apicomplexan parasite *Theileria* for ensuring its correct partitioning into the two daughter cells during host cell division (15). We also predicted occurrences of the SxIP and LYPxL motifs in RON8, which, together with RON2, RON4, and RON5, form part of the TJ (27). The presence of extra motif matches related to host cytoskeleton in TJ proteins indicates that the current model of motif-hijacking during this stage of host cell invasion is still incomplete and would benefit from our motif predictions. Furthermore, the identification of 4,822 motif matches in our data set in 66 proteins annotated as “hypothetical” in ToxoDB but with expression evidence (Fig. 3B) represents a potential avenue to explore their function.

On average, our pipeline predicted 82 motif matches per protein: 59 in micronemes, 77 in rhoptries, and 97 in dense granule proteins (Fig. 3A). While proteins, especially those with longer IDRs, such as dense granule proteins (Fig. S1A), are expected to have multiple motif occurrences, the relatively high number of predicted motif instances still points to some of those being likely non-functional. Most of the motif matches come from motif types that denote sites for PTMs, such as phosphorylation (Fig. 3A). This most likely results from very degenerate, i.e., unspecific, regular expressions available for motif types from the PTM class, such as ...[ST]...[ST], defining sites phosphorylated by GSK kinase. In regular expressions, a “.” corresponds to any amino acid possible at this position, and amino acids in brackets denote possible amino acids at this position in the motif. More than 2,000 motif matches alone were identified with this GSK kinase phosphorylation motif pattern. Phosphoproteomics data can be used to provide evidence for actual modification of these predicted motifs (53). Using available phosphosite data sets from ToxoDB, we found evidence of phosphorylation for ~6% of predicted phosphorylation sites (Fig. 3C and D), assigning higher confidence to these predicted motifs (Table S1). We generally observe that, for most motif classes, motif matches were identified only for a fraction of annotated motif types in ELM. For example, 38% of motif matches from the targeting class (TRG) correspond to diArg motifs, 35% of motif matches from the degradation class (DEG) correspond to SPOP degrons (see below), and 18% of predicted scaffolding motifs bind SH3 domains (Fig. S1B).

To further select for more likely functional motif matches, one can take into account functional aspects of the different motif types and the cellular context of the potentially secreted proteins (Fig. 3D). For example, as microneme proteins will be present in extracellular environments, motifs known to interact with domains from extracellular proteins would be expected to have more likely functional relevance. In our data set (Table S1), we found 401 motif matches in 48 microneme proteins from 12 motif types known to interact with extracellular domains. These predicted motif instances could be relevant during the recognition and attachment stages. Integrin binding is a common strategy for pathogens to attach to the host cell membrane (54–56). The most common integrin-binding motif is the RGD motif, which can bind to eight of the different alpha and beta integrin heterodimers (57). Within our data set, we found three motif matches of the RGD motif in three microneme proteins: MIC17C, Toxolysin 4 (TLN4), and chitinase-like protein CLP1. As some microneme proteins might be localized to the host cytosol, e.g., during host cell lysis and egress (58), we did not filter out matches from other cytosolic motif types in microneme proteins. Degradation signals, or degrons, are motifs recognized by E3 ubiquitin ligases and mark proteins for proteasomal degradation (59). Degrons might be relevant motif candidates for *Toxoplasma* infection, as other pathogens are known to modify protein levels via degrons to achieve outcomes, such as the stabilization of host components necessary for their survival (2) or modulating the activity of their proteins once inside the host (60). We found 475 degrons in 182 proteins: 73 in micronemes, 157 in rhoptries, and 245 in dense granule proteins. Speckle-type POZ protein (SPOP), an E3 ubiquitin ligase, recognizes its substrates via an ST-rich degron (61). We found 155 SPOP degron matches in 80 proteins. Since multivalency is a known mechanism by which SPOP substrates enhance their affinity to SPOP (61), one could prioritize the 34 secreted proteins containing two or more SPOP motif matches in our data set. Rhoptry protein toxolysin 1 (TLN1), a metalloprotease with a role in rhoptry maturation (62), but potentially detrimental for host establishment once secreted into the host cytosol, has 10 identical SPOP degron matches, which could be used to modulate TLN1 levels once within the host cytoplasm.

Another way to prioritize likely functional motifs that mediate binding to host proteins is by focusing on proteins with strong experimental evidence for secretion into the host cytoplasm. This excludes proteins known to participate in the biogenesis and maturation of the secretion organelles or that remain in the PV, and thus, would never come in contact with host proteins. Using our original data set (Table S1) and based on numerous studies on the subcellular localization of *Toxoplasma* rhoptry and dense granule proteins (22, 63–65), we selected 42 proteins with experimental evidence for secretion into or exposure to host subcellular compartments, including ROP1, ROP5, ROP18, GRA6, GRA16, and GRA24. By focusing on these proteins and excluding motifs that define sites for post-translational modifications, such as phosphorylation (MOD class in ELM), we defined a subset of 2,313 motif candidates (Table S2).

*Toxoplasma* is a eukaryotic organism; thus, it shares many motif-interacting domain types with its hosts. This means that some of our predicted *Toxoplasma* motifs might serve *Toxoplasma*-only functions and interact with other *Toxoplasma* proteins instead of host proteins. To further filter for motif matches that are more likely to mediate binding to host proteins, we can make use of the annotated taxonomic range of the motifs in ELM, through which we already annotated the presence of their interacting domain in either *Toxoplasma*, their potential hosts, or both. By focusing only on motifs interacting with domains present in mammals and birds, but not in *Toxoplasma*, and restricting ourselves further to the 42 proteins with strong secretion evidence, we reduced the number of motif matches with putative relevance in host infection to 725 (Table S2). Overall, these examples illustrate different strategies of how our resource can be further explored to prioritize motif candidates for experimental characterization. All additional annotations mentioned above are made available in Tables S1 and S2.

### Experimental validation of predicted TRAF6-binding motifs

For the experimental validation of predicted motifs, we focused on motifs that bind the tumor necrosis factor (TNF) receptor-associated factor 6 (TRAF6) protein. TRAF6 binds to proteins containing a PxExx[DE(*Φ*)] (*Φ* denotes hydrophobic residues) motif through its C-terminal MATH domain. TRAF6 plays important roles in immunity and development by contributing to the formation of various signaling protein complexes. During the innate immune response, TRAF6 is activated by interleukin one receptor (IL-1R), linking this receptor to the canonical activation of the transcription factor NF-κB (66). Through the addition of K63-linked polyubiquitin chains, TRAF6 primes proteins for complex assembly and not for proteasomal degradation. The TRAF6-binding motif is present in proteins, such as IL-1R, tumor necrosis factor protein CD40, and mitochondrial antiviral-signaling protein (MAVS) (67). In the context of infection, herpesviruses have been shown to use the TRAF6 motif-containing protein Tio to stimulate NF-κB activation (68).

**Fig 4:**
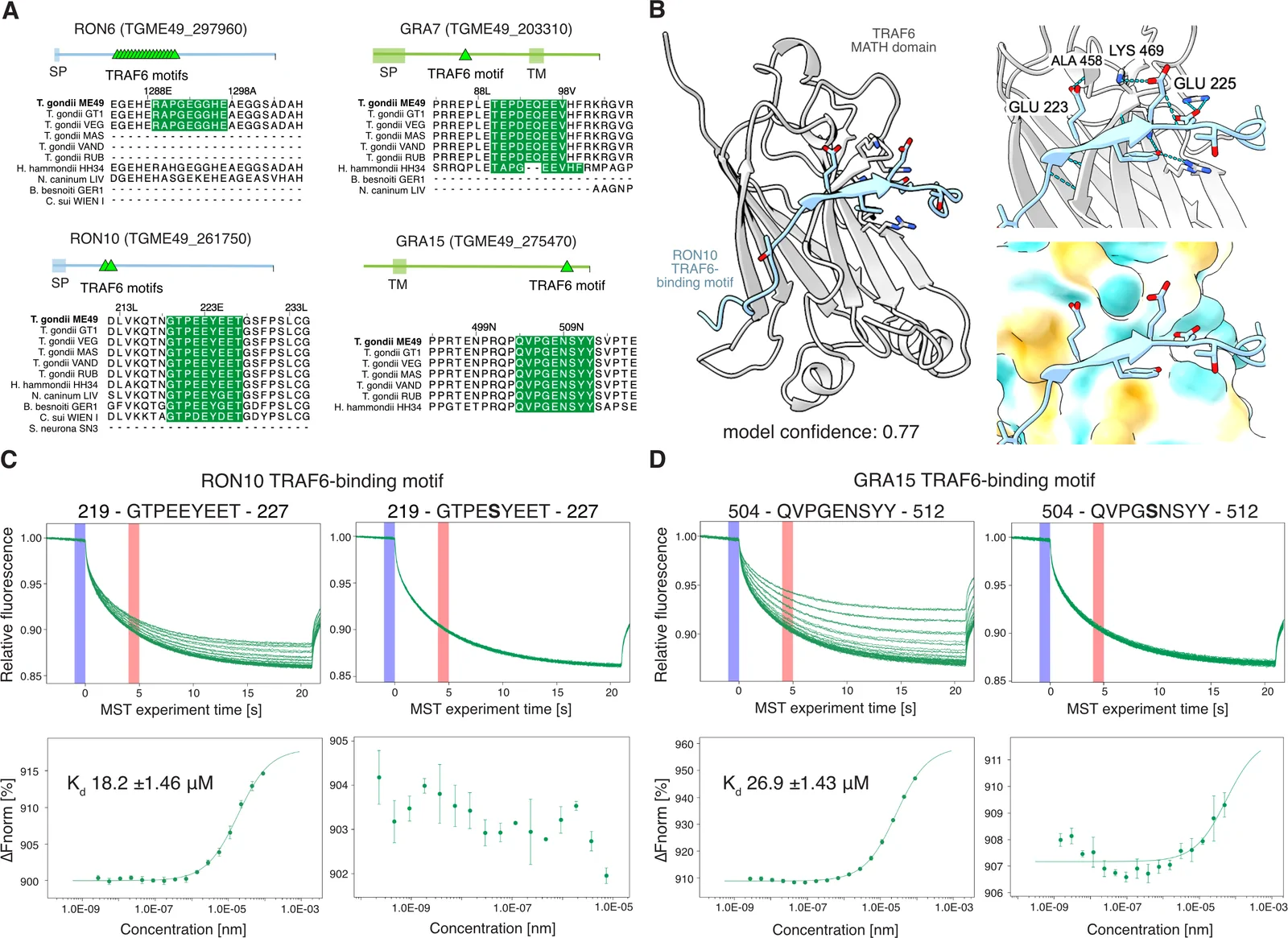
Experimental validation of TRAF6-binding motifs. (**A**) Protein diagrams of RON6, RON10, GRA7, and GRA15 indicating the position of predicted TRAF6-binding motifs with accompanying multiple sequence alignments showing their presence in different *Toxoplasma* strains and coccidian species. (**B**) AlphaFold model of TRAF6 MATH domain with TRAF6-binding motif from RON10. Right panels display the potential hydrogen bond network at the interaction interface (top) and secondary structure adopted by the TRAF6-binding motif (bottom). (**C**) RON10 TRAF6 motif tests. MST traces (top graph) and dose-response curves (bottom panel) of TRAF6 MATH domain binding to RON10 TRAF6 motif-containing peptide (left) and the E to S mutant (right). In the MST traces, the cold region is set at 0 s (blue) and the hot detection region at 5 s (red). (**D**) Same as in panel **C**, but for the GRA15 TRAF6 motif and the E to S motif mutant.

Among our predicted motif instances, we found 111 motif matches in 68 rhoptry and dense granule proteins. Given that the TRAF6-binding motif is known to appear multiple times within proteins (69), we hypothesized that the motif repetitions could interact with the TRAF6 trimer in a multivalent way. Following this criterion, we selected rhoptry neck protein RON6, which harbors 17 matches of the TRAF6-binding motif, most of them being identical repeats, and RON10, which harbors two different TRAF6-binding motif matches (Fig. 4A). RON10 is known to interact with RON9, but RON10 and RON9 do not have clear roles during infection (70). TRAF6 has been shown previously to interact with the dense granule proteins GRA7 and GRA15, which themselves play a role in modulating the innate immune response during *Toxoplasma* infection (71–74), and which are also present in our curated list of experimentally confirmed secreted proteins. However, the actual interaction interfaces of GRA7 and GRA15 with TRAF6 have not been described (25), making the TRAF6 motif matches in GRA7 and GRA15 interesting candidates for further experimental characterization (Fig. 4A).

We proceeded to determine the dissociation constants of peptides containing the TRAF6-binding motifs in RON6, RON10, GRA7, and GRA15 to the TRAF6 MATH domain using the microscale thermophoresis (MST) binding assay. To this end, we expressed and purified the human TRAF6 MATH domain and tested its binding against synthesized peptides containing the sequence of selected motif matches. In the case of RON6, we selected the most frequent version of its motif repeats. For RON10, we selected the second motif match that contained multiple negative charges in less conserved positions, which might contribute to binding (Fig. 4B). GRA7 and GRA15 only have one TRAF6-binding motif match each, which was selected for experimental testing. In addition, we ordered these wild-type peptide sequences with a glutamate to serine mutation at position three in the motifs, supposed to weaken or abrogate binding to the MATH domain because the conserved glutamate residue forms a hydrogen bond with backbone atoms of residue A458 in TRAF6 (Fig. 4B) (Table S3). Our experimental results confirm that the peptides containing the motifs from RON10 and GRA15 can bind to the TRAF6 MATH domain, and these interactions were lost when testing the mutant peptide versions. The dissociation constants for the RON10 (Kd 18.2 ± 1.46 µM) and GRA15 peptides (Kd 26.9 ± 1.43 µM) are as expected for functional motif interactions, in the lower micromolar range (Fig. 4C and D; Data S1, Table S3). These results represent the first evidence that RON10 could bind TRAF6, pointing to a role of RON10 in the regulation of the innate immune response.

## DISCUSSION

Our study represents the first comprehensive survey of short linear motifs in *Toxoplasma gondii* or any other apicomplexan parasite, yielding a data set of 24,097 motif matches within 295 proteins from *Toxoplasma* secretion organelles. There have been other efforts to predict and validate short linear motifs in pathogenic proteins (31, 75). However, they focused on only a subset of proteins or specific motifs, or other pathogens such as viruses (61). The ToxoDB resource offers single regular expression searches against any of the available parasite proteomes (45). However, this tool only handles a single regular expression at a time, and no further filtering of the motif matches to prioritize likely functional ones is provided, as our workflow does. Despite our efforts to further filter the large number of predicted motif matches, many likely non-functional motif matches remain in our data set. More sophisticated computational approaches for match filtering could make use of machine learning on larger sets of validated motif instances, which, at the moment, are not available for *Toxoplasma* and other parasites. Conservation of motif matches is often used as an additional line of evidence for the prioritization of likely functional motif matches (76). Unfortunately, *Toxoplasma*-secreted proteins tend to be more novel, less conserved, and display longer regions of disorder (Fig. S1A) (77). This makes it difficult to build good sequence alignments to compute common motif conservation metrics like in (76). Nevertheless, we included the assessment of the presence of our motif matches in different *Toxoplasma* strains and the few closely related parasite species as supplementary conservation metrics (Table S1), which could be useful when working with different strains or in understanding motif variation and evolution.

Our data set includes proteins located in secretory organelles that might not get secreted to host cellular compartments, e.g., proteins involved in the biogenesis of secretory organelles and the maturation of their proteins, and thus, predicted motifs in these proteins will not function in mediating binding to host proteins. We decided to retain these motif matches in our resource, since they might still mediate binding to other *Toxoplasma* proteins, and thus can serve as starting points for the characterization of these proteins that often have unknown functions. However, we provide multiple annotations that further enable users to prioritize proteins and motif matches with higher probability to mediate interactions with host proteins.

It has been previously speculated that *Toxoplasma* induces NF-κB activation and macrophage recruitment to limit tissue invasion, assuring host survival and promoting development into the chronic bradyzoite form (50). The experimentally tested peptides containing the TRAF6-binding motifs in RON10 and GRA15 might support this process by connecting TRAF6-dependent IL-1 signaling with NF-κB activation. However, proving the functional relevance of these motifs as well as the binding of RON10 and GRA15 to TRAF6 in physiological settings would require further experimentation. Despite GRA7 being known to interact with TRAF6, we did not see any binding of the peptide containing its TRAF6 motif. TRAF6 and GRA7 could still interact through a different interface or through a third binding partner. Even though the RON6 peptide did not show binding to the TRAF6 MATH domain, it might still display binding in cells via multivalent interactions involving its 17 putative TRAF6-binding motif matches and TRAF6 trimers.

Our systematic approach for predicting instances of known motif types in *Toxoplasma* proteins indicates that there might be a substantial number of motifs in *Toxoplasma* to be characterized. The nature of our discovery pipeline allows for the discovery and characterization of motifs involved in housekeeping Toxoplasma cellular processes, and not during infection. *Toxoplasma*, as a eukaryote, has many domain types that it shares with its hosts, and which are able to interact with motifs that participate in endogenous processes for parasite motility, cytoskeletal rearrangements, and parasite development. In this study, we primarily focused on the infection process, but the discovery and identification of Toxoplasma motifs functioning only in internal parasite processes is also an important and exciting research area, exemplified by the TEXEL export motifs key to the appropriate export of GRA proteins (42). Thus, this data set represents a valuable resource for hypothesis generation and design of downstream experiments to also study molecular mechanisms of *Toxoplasma gondii* infection and biology. Structural models of predicted domain-motif interactions could further be used to design disruptive mutations to validate predicted modes of binding (4). Identified and experimentally validated pathogen-host motif-domain interactions can form the basis for the development of therapeutic strategies aimed at perturbing these interactions and thereby interfering with infection processes (4, 10). Motif discovery in pathogens remains a much underexplored area of research but holds great promise for improved understanding of infection mechanisms and the better design of effective treatments against parasite infection.

## MATERIALS AND METHODS

### Retrieval of sequence, structure, protein localization, and motif data

*Toxoplasma* strain proteomes, as well as those of five closely related organisms from the Sarcocystidae clade, were retrieved from the ToxoDB resource (45). The FASTA files of the annotated proteomes were collected for the following organisms: *Toxoplasma gondii* GT1 (Type I), *T. gondii* MAS, and *T. gondii* ME49 (Type II and reference strain), *T. gondii* RUB, and *T. gondii* VEG (Type III), *T. gondii* VAND, *Hammondia hammondii, Neospora caninum, Besnoitia besnoitii, Cystoisospora suis* Wien I, and *Sarcosystis neurona* SN3. Using the ToxoDB search strategies, we retrieved mass spectrometry proteomic peptide mapping data, phosphosites, and cellular location predictions from the HyperLOPIT experiment (44) for the proteins of *T. gondii* ME49. All available AlphaFold database (AFDB) (78) predicted structures for *Toxoplasma gondii* ME49 were retrieved from the Google Cloud Console public-datasets-deepmind-alphafold-v4, taxonomy ID: 50877 (02.05.2025). The latest data set of motif classes was downloaded from the ELM resource (30.04.2025) (26). The file contained each motif code name, its regular expression (REGEX), and supplementary information. We did manual annotation of the motif classes to determine if their taxonomic range included vertebrates and *Toxoplasma*, i.e., if the domain was annotated in ELM to be present in the respective organism for interaction with the motif, ultimately excluding motif classes of organisms such as plants, insects, and fungi. We also added a manual annotation on whether the motif is functional in an extracellular context and if it requires phosphorylation or modification to interact with its domain partner. We retrieved available *T. gondii* domain mappings from UniProt (29.07.2025) (51), which came from Pfam, PROSITE, and SMART. To define our data set of 42 confirmed secreted proteins, a literature review in the following articles was done, and proteins from rhoptries and dense granules with available experimental evidence of being in contact with host components were retained (22, 58, 63–65).

### Motif prediction and structural assessments

We determined the presence of motif matches in *T. gondii* ME49 by doing a baseline REGEX search. To assess the accessibility of these motif predictions, two approaches were selected: one that takes into account the intrinsic disorder of the sequence and another that evaluates the accessibility of the motif from predicted AlphaFold DB (AFDB) structures. The IUPred3 software (47) was used to calculate the disorder probability of the full proteome of *T. gondii* ME49. Once a REGEX match was determined, the IUPred3 score from all motif residues was retrieved and averaged. Complementarily, using available AFDB structures, we took the pLDDT scores from all motif residues and averaged them (79). We calculated residue accessibility using Dictionary of Secondary Structure of Proteins (DSSP) software (80) on each AFDB structure. The individual residue scores were then standardized according to reference 81 to obtain relative surface accessibility scores (RSA). We then took the RSA scores of all motif residues and averaged them. We determined if a motif match overlapped with known domain boundaries from UniProt by using positional information.

### Motif organism presence assessment

A reciprocal best hit search was carried out using BLAST to construct sets of orthologous sequence groups among *Toxoplasma* strains and related species. These sequence groups contained the sequences with the lowest e-value from each organism’s proteome, so alignments contained a standard number of sequences and were easily comparable. Multiple sequence alignments were produced for each sequence group using ClustalO with default parameters (82). Standard conservation measurements (76) were not possible as our alignments contained only a few species. Thus, the conservation of the motifs was approximated by carrying out a presence assessment. A motif was considered present in another organism if a match of the same class was present in its sequence within a window of 15 amino acids in the alignment around the start site of the motif match of the reference *Toxoplasma* strain ME49, and then the presence proportion of the total number of both strains and species was calculated and associated with each motif match, resulting in three different scores: the proportion of strains, species, and overall organisms.

### Data enrichment and filtering

The data from the ELM REGEX motif matches, their conservation, IUPred3, pLDDT, and accessibility scores were combined into a single table together with their respective protein information on subcellular location, annotations, domain overlap, and modifications. This was carried out using their unique ToxoDB identifiers and the motif name, match number, and residue positions. Data filtering to define a set of motif matches was carried out with three different strategies. First, we retained motifs from proteins with at least one peptide mapped to them from retrieved mass spectrometry data, to make sure proteins were expressed. We then made use of the ELM taxonomy range by ensuring that the taxonomic range of the motif instance, if the motif has been reported in a certain group of organisms, was valid for the *Toxoplasma* matches. Only motif classes that apply to vertebrates and apicomplexans were kept, mainly excluding those characterized in plants, insects, or fungi. The classes valid in vertebrates were kept if the motifs were located in secreted proteins, as they would be in contact with the vertebrate host (human, a mammal, or birds) cell components. The classes only valid in extracellular contexts were kept if the motifs were located in microneme proteins, as among secreted proteins, they would be be the ones in this location. We applied structural filters in two steps; only motif matches with IUPred3 scores above 0.4 were considered, following disordered region filters in (83). Second, for motifs in proteins with available AFDB structures, we retained the ones with a pLDDT ≤ 80 (79) and an average RSA ≥ 0.25 (84). For motifs without AFDB structures, we retained only the ones outside of known domain boundaries.

### AlphaFold modeling

The TRAF6 MATH domain and the RON10 TRAF6-binding motif were selected for structural modeling. We used a local installation of AlphaFold Multimer version 2.3.219 (85). We manually defined domain boundaries to include missing secondary structure with high pLDDT values. We extended the motif sequence by including five residues at each sequence end. Structural model visualizations were produced using ChimeraX-1.10 (86).

The local installation of AlphaFold Multimer version 2.3.2 was run using the following parameters and following Alphafold GitHub instructions (https://github.com/deepmind/alphafold#running-alphafold):

--model_preset = multimer \

--db_preset = full_dbs \

--max_template_date = 2020-05-14 \

--num_multimer_predictions_per_model = 1 \

--use_gpu_relax = True \

--bfd_database_path=/mnt/storage/alphafold/v232/bfd/bfd_metaclust_clu_complete_id30_c90_final_seq.sorted_opt \

--mgnify_database_path=/mnt/storage/alphafold/v232/mgnify/mgy_clusters_2022_05.fa \

--obsolete_pdbs_path=/mnt/storage/alphafold/v232/pdb_mmcif/obsolete.dat \

--pdb_seqres_database_path=/mnt/storage/alphafold/v232/pdb_seqres/pdb_seqres.txt \

--template_mmcif_dir=/mnt/storage/alphafold/v232/pdb_mmcif/mmcif_files \

--uniprot_database_path=/mnt/storage/alphafold/v232/uniprot/uniprot.fasta \

--uniref90_database_path=/mnt/storage/alphafold/v232/uniref90/uniref90.fasta \

--uniref30_database_path=/mnt/storage/alphafold/v232/uniref30/UniRef30_2021_03 \

--use_precomputed_msas = True

### Protein expression and purification

A gene encoding the human TRAF6 C-domain (residues 346-504, UniProt ID Q9Y4K3) was synthesized (GeneArt, Thermo Fisher Scientific) and subcloned into the pCoofy4 plasmid (87) containing an N-terminal His6-MBP-3C fusion tag. For the human TRAF6 C-domain (InterPro: IPR008974) gene, the native DNA sequence was used. The resulting bacterial expression construct was freshly transformed into *E. coli* BL21(DE3) codon + RIL cells (Stratagene). Precultures were grown overnight at 37°C in LB medium supplemented with 30 µg/mL kanamycin and 35 µg/mL chloramphenicol and used to inoculate the large-scale expression cultures. Then, 10 mL preculture was added to 1 L of TB-FB medium supplemented with 30 µg/mL lanamycin and 35 µg/mL chloramphenicol. The cultures were grown at 37°C until the OD600 reached ~0.8, after which the temperature was reduced to 18°C, and expression of the hTRAF6 C-domain was induced with 0.5 mM IPTG. After overnight expression at 18°C, the cultures were harvested by centrifugation (30 min, 5000 g, 4°C), and the pellets were flash-frozen in liquid nitrogen and stored at −80°C until the start of the protein purification.

The cells were lysed by five passages through a microfluidizer device, followed by centrifugation (30 min, 140,000 × *g*, 4°C). The cleared lysate was loaded onto a 5-mL Protino Ni-NTA column (Macherey-Nagel) pre-equilibrated with 50 mM Tris-HCl, pH 8.0, 500 mM NaCl, and 20 mM imidazole. After loading, the Ni-NTA column was washed with equilibration buffer until the A280-nm signal returned to baseline and eluted in a 60-mL gradient going from 0 to 100% of Ni-NTA elution buffer (50 mM Tris-HCl, pH 8.0, 500 mM NaCl, 350 mM imidazole). The Ni-NTA elution fractions were analyzed by SDS-PAGE, and the fractions containing the His6-MBP-3C-hTRAF6 C-domain fusion protein were pooled. Then, 2 mg of His6-tagged HRV 3C protease was added, and the sample was dialyzed overnight at 4°C to IEX buffer (25 mM HEPES, pH 7.5, 100 mM NaCl, 20 mM imidazole). The next day, the dialyzed sample was loaded onto a 5-mL Protino Ni-NTA column (Macherey-Nagel) coupled to a 5-mL HiTrap Q HP anion exchange column (Cytiva), both equilibrated with IEX buffer. The untagged hTRAF6 C-domain protein was present in the flow-through of the coupled columns, which was concentrated to 5 mL and injected into a HiLoad 16/600 Superdex 75 pg size exclusion chromatography column (Cytiva) pre-equilibrated with 25 mM HEPES pH 7.5, 150 mM NaCl, and 1 mM DTT. The elution fractions containing pure hTRAF6 C-domain protein were pooled and concentrated to 2.1 mg/mL (111 µM). The final hTRAF6 C-domain protein samples were aliquoted, flash-frozen in liquid nitrogen, and stored at −80°C until further use. The identity of the hTRAF6 C-domain protein was verified by mass spectrometry (EMBL Proteomics Core Facility), and the oligomerization state (monomeric) and absence of aggregates were confirmed by size exclusion chromatography column coupled to multi-angle light scattering (SEC-MALS). The stability of the hTRAF6 C-domain protein in the storage buffer was assessed by nano-Differential Scanning Fluorimetry (nano-DSF; Tm ~46.8°C).

### Binding assays

We selected four motifs from four different *Toxoplasma* proteins with different AF model scores for carrying out binding assays as described in the Results, together with the human MAVS TRAF6 motif, being selected as a control (67). Peptides of 15 amino acids containing the motifs were defined, as well as versions containing E > S point mutations at motif position + 3. The peptides were labeled with 5-carboxyfluorescein (5-Fluo) at the C-terminus by Biosyntan GmbH (https://www.biosyntan.de) to carry out microscale thermophoresis (MST). The lyophilized peptides were resuspended in 25 mM HEPES, pH 7.5, and 150 mM NaCl to a concentration of 2 mM, and the pH was adjusted to 7.5 when necessary. MST measurements were performed using a Monolith NT.115 (NanoTemper Technology). Twofold serial dilutions of the hTRAF6 protein (99 μM) were mixed in a 1:1 ratio with 100 nM peptides. Titration measurements were taken for all *Toxoplasma* peptides, and the human MAVS control, with two-fold serial dilutions of the hTRAF6 protein (99 μM) mixed in a 9:1 ratio with 500 nM. All measurements were performed in triplicate and carried out at 25°C with an LED excitation power of 20% and a medium MST power. Finally, the data were analyzed assuming a 1:1 binding model and graphed using the MO.Affinity Software (NanoTemper Technology).

## Supplementary Material

Reviewer comments

## Data Availability

The motif discovery pipeline, processing, and analysis code is made available on GitHub (https://github.com/JesnsAV/Toxo_Motifs).

## SUPPLEMENTAL MATERIAL

Supplemental figure legends spectrum.00724-26-s0001.docx Legends for Data S1, Fig. S1, and Tables S1 and S2.

Data S1 spectrum.00724-26-s0002.pdf MST extended results.

Fig. S1 spectrum.00724-26-s0003.tiff Protein disorder content in different subcellular compartments and summary statistics of motif matches per motif class.

Table S1 spectrum.00724-26-s0004.txt Motif predictions in microneme, rhoptry, and dense granule proteins.

Table S2 spectrum.00724-26-s0005.txt Motif predictions in 42 experimentally tested secreted rhoptry and dense granule proteins.

Table S3 spectrum.00724-26-s0006.xlsx Selected TRAF6 motif-containing peptides and binding assay results.

OPEN PEER REVIEW reviewer-comments.pdf An accounting of the reviewer comments and feedback.

ASM does not own the copyrights to Supplemental Material that may be linked to, or accessed through, an article. The authors have granted ASM a non-exclusive, world-wide license to publish the Supplemental Material files. Please contact the corresponding author directly for reuse.
