# Supplementary material for: Short linear motifs—underexplored players driving Toxoplasma gondii infection
Source: Microbiol Spectr. 2026 Aug 5;14(9):e00724-26. doi: 10.1128/spectrum.00724-26 (PMC13532356; doi:10.1128/spectrum.00724-26)
Supplement: Supplementary file 1 [file reviewer-comments.pdf]

ASM Microbiology Spectrum

Short linear motifs - Underexplored players driving Toxoplasma gondii infection

Jesus Alvarado Valverde, Karine Lapouge, Arne Boergel, Kim Remans, Katja Luck, and Toby Gibson

Corresponding Author(s): Jesus Alvarado Valverde, European Molecular Biology Laboratory

Review Timeline:

Submission Date:	March 16, 2026
Editorial Decision:	April 27, 2026
Revision Received:	June 26, 2026
Accepted:	July 10, 2026

Editor: Prakash Srinivasan

Reviewer(s): Disclosure of reviewer identity is with reference to reviewer comments included in decision letter(s). The following individuals involved in review of your submission have agreed to reveal their identity: Julia D Romano (Reviewer #2)

Transaction Report:

(Note: With the exception of the correction of typographical or spelling errors that could be a source of ambiguity, letters and reports are not edited. The original formatting of letters and referee reports may not be reflected in this compilation.)

DOI: https://doi.org/10.1128/spectrum.00724-26

Re: Spectrum00724-26 (**Short linear motifs - Underexplored players driving Toxoplasma gondii infection**)

Dear Dr. Jesus Alvarado Valverde:

Thank you for the privilege of reviewing your work. Below you will find my comments, instructions from the Spectrum editorial office, and the reviewer comments.

The reviewers recognize the potential value of your study but have identified issues that should be addressed to improve clarity and interpretation. We invite you to submit a revised manuscript that responds to these comments.

Please return the manuscript within 60 days; if you cannot complete the modification within this time period, please contact me. If you do not wish to modify the manuscript and prefer to submit it to another journal, notify me immediately so that the manuscript may be formally withdrawn from consideration by Spectrum.

Revision Guidelines
To submit your modified manuscript, log into the submission site at https://spectrum.msubmit.net/cgi-bin/main.plex. Go to Author Tasks and click the appropriate manuscript title to begin. The information you entered when you first submitted the paper will be displayed; update this as necessary. Note the following requirements:

• Upload point-by-point responses to the issues raised by the reviewers in a file named "Response to Reviewers," NOT in your cover letter.
• Upload a compare copy of the manuscript (without figures) as a "Marked-Up Manuscript" file.
• Upload a clean .DOC/.DOCX version of the revised manuscript and remove the previous version.
• Each figure must be uploaded as a separate, editable, high-resolution file (TIFF or EPS preferred), and any multipanel figures must be assembled into one file.
• Any supplemental material intended for posting by ASM should be uploaded with their legends separate from the main manuscript. You can combine all supplemental material into one file (preferred) or split it into a maximum of 10 files with all associated legends included.

For complete guidelines on revision requirements, see our Submission and Review Process webpage. Submission of a paper that does not conform to guidelines may delay acceptance of your manuscript.

Data availability: ASM policy requires that data be available to the public upon online posting of the article, so please verify all links to sequence records, if present, and make sure that each number retrieves the full record of the data. If a new accession number is not linked or a link is broken, provide Spectrum production staff with the correct URL for the record. If the accession numbers for new data are not publicly accessible before the expected online posting of the article, publication may be delayed; please contact production staff (Spectrum@asmusa.org) immediately with the expected release date.

Publication Fees: For information on publication fees and which article types are subject to charges, visit our website. If your manuscript is accepted for publication and any fees apply, you will be contacted separately about payment during the production process; please follow the instructions in that e-mail. Arrangements for payment must be made before your article is published.

ASM Membership: Corresponding authors may join or renew ASM membership to obtain discounts on publication fees. Need to upgrade your membership level? Please contact Customer Service at Service@asmusa.org.

The ASM Journals program strives for constant improvement in our submission and publication process. Please tell us how we can improve your experience by taking this quick Author Survey.

Thank you for submitting your paper to Spectrum.

Sincerely,
Prakash Srinivasan
Editor
Microbiology Spectrum

Reviewer #1 (Comments for the Author):

This manuscript by Valverde et al, presents the first comprehensive survey of short linear motifs (SLiMs) in Toxoplasma gondii secreted proteins. The authors curate 21 experimentally validated motif instances, develop a computational prediction pipeline yielding 24,291 motif matches across 295 secreted proteins, and experimentally validate TRAF6-binding motifs in RON10 and GRA15 using microscale thermophoresis. The topic is timely and the resource has potential value for the field. However, the

paper suffers from systematic overstatement of its findings, a fundamental conceptual gap in its biological framing, and several interpretive problems. A focused reanalysis or supplementary analysis restricted to proteins with confirmed host-cell delivery would substantially increase the impact of the work.

Major Comments

1. The authors state they focused on proteins "secreted into host cells," yet the vast majority of the 295 proteins in the dataset have no experimental evidence of reaching the host cytoplasm or nucleus. Many dense granule proteins reside in the PV lumen or are embedded in the PVM with no cytoplasmic face exposed to the host. Microneme proteins function extracellularly during invasion. Including these proteins in a dataset framed around host-pathogen motif interactions is misleading. The authors should either restrict their analysis to proteins with confirmed or strongly supported evidence of host-cell delivery, or at minimum provide a clear supplementary categorization distinguishing proteins by their level of host-delivery evidence, and revise the framing throughout to reflect that most predictions concern candidate rather than confirmed host-interaction proteins.
2. Unlike viruses and bacteria, Toxoplasma is itself a eukaryote and uses the same regulatory motif machinery internally. A predicted SH3-binding motif or NLS in rhoptry or dense granule protein could equally serve endogenous parasite functions such as organelle biogenesis, intracellular trafficking, parasite cell cycle regulation. The authors acknowledge this briefly but never resolve it, and the entire computational framework assumes that motifs in secreted proteins are there to interact with host proteins. This assumption is unjustified for the broad protein set used, and it is a conceptual problem that distinguishes this work from the viral and bacterial motif mimicry literature the authors cite as precedent.
3. The most scientifically coherent application of this pipeline would have been a focused analysis restricted to proteins with strong evidence of host cytoplasmic or nuclear delivery, the nuclear-targeted effectors GRA16, GRA24, and the growing set of dense granule proteins confirmed to cross the PVM. For these proteins, the host compartment is defined, the relevant binding partners are partially known, and the eukaryote-eukaryote ambiguity problem is substantially reduced because predicted host-nuclear motifs are unlikely to be serving parasite-internal functions. Such a focused analysis would have produced a cleaner signal-to-noise ratio, immediately actionable hypotheses, and a more convincing demonstration of the pipeline's utility. The current approach yields 24,291 predictions that are nominally a resource but in practice require so much additional biological filtering that their utility for any given research question is limited. The authors should consider whether a focused reanalysis of confirmed host-delivered effectors could be incorporated, or at minimum should reframe the resource accordingly and explicitly recommend this as the primary use case.
4. The Importance section states that the authors "experimentally test proteins containing a motif involved in the innate immune response." This is an overstatement. Only synthetic 15-amino acid peptides were tested for binding to a purified recombinant domain in vitro. The full-length proteins were not tested; the native infection context was not recapitulated. This distinction must be reflected consistently throughout the manuscript - in the Abstract, Importance section, Results, and Discussion. The peptide binding results are encouraging and appropriate as proof-of-concept, but they do not constitute evidence that these motifs drive host-pathogen interactions during infection.
5. GRA7 has been shown to interact with TRAF6 (Yang et al., 2016, PMID: 26553469), yet the TRAF6-binding motif peptide from GRA7 showed very weak binding. This is a significant problem given that GRA7-TRAF6 interaction is one of the motivating examples for the entire experimental section. The authors do not address this discrepancy.
6. The ELM resource predicts PEXEL motifs for Toxoplasma proteins, but it has been established for nearly a decade that TgASP5 cleaves a TEXEL motif (RRLxx)
that is distinct from the Plasmodium PEXEL (RxLxE/Q/D). The authors use ELM's PEXEL annotation without flagging this error, which is a factual inaccuracy in the curation and undermines confidence in the quality of the motif annotation more broadly.
7. The Discussion opens with speculative biological interpretation of the RON10-TRAF6 interaction and its potential role in NF-κB signaling and bradyzoite development. This framing presents the RON10 finding as the paper's headline result, when the primary contribution of the work is actually the curated motif dataset and prediction pipeline.

Minor Comments

1. The statement "all these secreted proteins interact with host proteins throughout the infection cycle" (lines 113-115) is an overstatement and should be revised to reflect that only a subset of secreted proteins have demonstrated host interactions.
2. The claim that parasite protein export uses host machinery (lines 197-198) requires a specific citation supporting host machinery involvement in Toxoplasma effector translocation.

Reviewer #2 (Comments for the Author):

Many intracellular pathogens secrete effector proteins that interact with host proteins, often through short linear motifs on the pathogen proteins that bind to structured domains in host proteins. Identifying these motifs in secreted parasite proteins can provide valuable insight into their mechanistic functions. In this study, the authors created a computational discovery pipeline designed to mine databases for short linear motifs within the secretome of the apicomplexan parasite Toxoplasma gondii.

Identifying motifs in parasite proteins, particularly those with unknown functions, is a worthwhile approach that has previously led to important functional insights. For example, characterization of the GRA14 protein revealed TSG101 and ALIX-interacting motifs, which led to the discovery of its role in recruiting host ESCRT machinery to the parasitophorous vacuole (PV). In that case and others, the identification of motifs was performed on a single protein case. The pipeline employed in this study identified a number of potentially interesting motifs across the predicted secretome Toxoplasma.

However, the manuscript would benefit from a more detailed description of both the pipeline and the resulting analyses. The inclusion of in vitro binding assays to test predicted motifs is a strength. That said, these assays rely on short synthetic peptides (15 amino acids) interacting with a purified domain of TRAF6 known to bind the motif, instead of the full-length protein. This approach does not fully capture the biological context in which these interactions occur. A more informative experiment would be to use the peptides in pull-down assays with full-length TRAF6 from cellular lysates. Ideally, co-immunoprecipitation experiments would provide the most physiologically relevant validation of these predicted interactions.

Specific comments:
Line 77: Including one or two examples of interactions between either virial or secreted bacterial effectors and host proteins, mediated by short linear motifs, would be informative.

Lines 109-110: Dense granules are not membraneless organelles. They initially bud from the Golgi and are surrounded by a lipid bilayer. This description should be corrected.

Line 113: The statement, "All these proteins interact with host proteins throughout the infection cycle..." should be qualified. While this may be true for the parasite proteins that are inserted into the PV membrane and are exported into the host cell, it may not necessarily be true of the parasite proteins confined to the PV lumen (line111). Please rephrase for accuracy.

Line 135: Please include the URL for the Eukaryotic Linear Motif (ELM) reference.

Lines 139-144: Please provide additional detail on motif classification and ELM classes. Specifically:
• Identify which two Toxoplasma motif instances were previously annotated (DOC), including motifs and corresponding protein.
• For the eleven novel motif instances, please list the motifs and proteins and explain the rationale for assigning them to the scaffolding class in ELM.
• Please list the eight additional novel motif instances and their associated proteins. Is there anything else known about these motifs? How were they identified if they could not be directly annotated using ELM?

Lines 176-177: The description of GRA14 function should be expanded to note that recruitment of ESCRT (ALIX and TSG101) contributes not only to host cytosolic material uptake via PV membrane budding, but also to the internalization of host Rab vesicles.

Table 1 (lines 179-181): Please define the ELM motif abbreviations used in the table.

Line 190: Clarify what is meant by "during establishment".

Line 193-194: Specify the motif from GRA24 that binds to MAPK14

Lines 196-198: The description of protein export is unclear and appears to conflate distinct processes. Please distinguish between the export of parasite proteins from the PV, which relies on parasite machinery, and the trafficking of parasite proteins within the host cell, such as to the nucleus, which uses host machinery.

Lines 222-224: A clearer and more detailed description of the computational pipeline is needed. Clarify whether this refers to a novel algorithm or a workflow built on existing database search tools. For the ELM database searches using "regular expressions defined for annotated motif classes" please specify which expressions were used.

We thank both reviewers for their highly constructive and to-the-point requests made on our manuscript. As described below, we tried to incorporate most of the suggestions made, and we think that, as a result, the manuscript substantially improved in clarity and quality.

Reviewer #1 (Comments for the Author):

This manuscript by Valverde et al, presents the first comprehensive survey of short linear motifs (SLiMs) in Toxoplasma gondii secreted proteins. The authors curate 21 experimentally validated motif instances, develop a computational prediction pipeline yielding 24,291 motif matches across 295 secreted proteins, and experimentally validate TRAF6-binding motifs in RON10 and GRA15 using microscale thermophoresis. The topic is timely and the resource has potential value for the field. However, the paper suffers from systematic overstatement of its findings, a fundamental conceptual gap in its biological framing, and several interpretive problems. A focused reanalysis or supplementary analysis restricted to proteins with confirmed host-cell delivery would substantially increase the impact of the work.

Major Comments

1. The authors state they focused on proteins "secreted into host cells," yet the vast majority of the 295 proteins in the dataset have no experimental evidence of reaching the host cytoplasm or nucleus. Many dense granule proteins reside in the PV lumen or are embedded in the PVM with no cytoplasmic face exposed to the host. Microneme proteins function extracellularly during invasion. Including these proteins in a dataset framed around host-pathogen motif interactions is misleading. The authors should either restrict their analysis to proteins with confirmed or strongly supported evidence of host-cell delivery, or at minimum provide a clear supplementary categorization distinguishing proteins by their level of host-delivery evidence, and revise the framing throughout to reflect that most predictions concern candidate rather than confirmed host-interaction proteins.

We revised the entire manuscript to make it clearer that the list of proteins we focus on has some probability of being secreted into host cellular compartments or being otherwise in contact with host proteins, but that this has not been firmly experimentally supported. We would like to point out that microneme proteins, although secreted to the extracellular space, can interact with extracellular host proteins such as receptors and peptidases, which is why we included microneme proteins in our list and examples (lines 291-302). We used the hyperLOPIT dataset to obtain information on the localisation of Toxoplasma proteins, and based on this dataset, also retained proteins from rhoptries and dense granules potentially found to locate to the parasitophorous vacuole membrane and the cyst membrane, since transmembrane proteins can, in part, be exposed to the host cytosol and thus interact with host proteins as well. We added an explanation and more details to our initial selection of proteins (lines 209-220). In addition, we manually curated 42 proteins with strong experimental evidence for secretion into the host cytosol from rhoptries and dense granules, and flagged those in our entire dataset and provided them also as a separate supplementary table (Table S2) for readers to focus on. We also discuss this aspect in the discussion (413-420) and extended the Methods section regarding the curation of the additional dataset of 42 proteins (lines 491-494).

2. Unlike viruses and bacteria, Toxoplasma is itself a eukaryote and uses the same regulatory motif machinery internally. A predicted SH3-binding motif or NLS in rhoptry or dense granule protein could equally serve endogenous parasite functions such as organelle biogenesis, intracellular trafficking, parasite cell cycle regulation. The authors acknowledge this briefly but

never resolve it, and the entire computational framework assumes that motifs in secreted proteins are there to interact with host proteins. This assumption is unjustified for the broad protein set used, and it is a conceptual problem that distinguishes this work from the viral and bacterial motif mimicry literature the authors cite as precedent.

We have revised the manuscript to make these points clearer. We had also previously annotated all predicted motifs for whether the interacting domain type can occur in host proteins only or also in Toxoplasma as an additional possibility to filter our predictions, and we now make this clearer in the text (lines 329-338, Table S1, S2). While the manuscript has put the main focus on the prediction of Toxoplasma motifs that likely mediate binding to host proteins, we now make it clearer in the manuscript that other motifs, only relevant for Toxoplasma functions, might be of interest to study Toxoplasma biology why we kept them in the dataset (lines 416-420, 435-445). We would like to point out that SH3 domains are absent in the Toxoplasma proteome, representing an interesting example of motif matches that are more likely to be of relevance for interaction with host proteins.

3. The most scientifically coherent application of this pipeline would have been a focused analysis restricted to proteins with strong evidence of host cytoplasmic or nuclear delivery, the nuclear-targeted effectors GRA16, GRA24, and the growing set of dense granule proteins confirmed to cross the PVM. For these proteins, the host compartment is defined, the relevant binding partners are partially known, and the eukaryote-eukaryote ambiguity problem is substantially reduced because predicted host-nuclear motifs are unlikely to be serving parasite-internal functions. Such a focused analysis would have produced a cleaner signal-to-noise ratio, immediately actionable hypotheses, and a more convincing demonstration of the pipeline's utility. The current approach yields 24,291 predictions that are nominally a resource but in practice require so much additional biological filtering that their utility for any given research question is limited. The authors should consider whether a focused reanalysis of confirmed host-delivered effectors could be incorporated, or at minimum should reframe the resource accordingly and explicitly recommend this as the primary use case.

We thank the reviewer for the thoughtful suggestions. We have revised the manuscript to provide clearer examples for how the large number of predicted motif matches can be further filtered (lines 317-327), and we provide the annotations needed for these filtering steps (Table S1, S2). We also explain why providing the whole dataset might still be useful for some researchers who study Toxoplasma proteins of unknown function, either with an interest in the infection process or in Toxoplasma biology (lines 435-445).

4. The Importance section states that the authors "experimentally test proteins containing a motif involved in the innate immune response." This is an overstatement. Only synthetic 15-amino acid peptides were tested for binding to a purified recombinant domain in vitro. The full-length proteins were not tested; the native infection context was not recapitulated. This distinction must be reflected consistently throughout the manuscript - in the Abstract, Importance section, Results, and Discussion. The peptide binding results are encouraging and appropriate as proof-of-concept, but they do not constitute evidence that these motifs drive host-pathogen interactions during infection.

We have rephrased all statements that we tested motifs or proteins and indicated specifically that we only tested peptides containing the motifs across the abstract (lines 31-32), importance (lines 48-49), results (line 371) and discussion (line 424) sections.

5. GRA7 has been shown to interact with TRAF6 (Yang et al., 2016, PMID: 26553469), yet the TRAF6-binding motif peptide from GRA7 showed very weak binding. This is a significant problem given that GRA7-TRAF6 interaction is one of the motivating examples for the entire experimental section. The authors do not address this discrepancy.
We are now discussing a possible reason for this negative result in the discussion section (lines 428-430).

6. The ELM resource predicts PEXEL motifs for Toxoplasma proteins, but it has been established for nearly a decade that TgASP5 cleaves a TEXEL motif (RRLxx) that is distinct from the Plasmodium PEXEL (RxLxE/Q/D). The authors use ELM's PEXEL annotation without flagging this error, which is a factual inaccuracy in the curation and undermines confidence in the quality of the motif annotation more broadly.
We thank the reviewer for making this important point. We have removed the PEXEL predictions from our datasets. We initially included it due to its high similarity in sequence and because the ELM resource does not have a class for the TEXEL motif. We also checked all other motif types from the ELM resource, and we had already excluded other motif classes that are irrelevant to Toxoplasma or host biology, such as plant or fungus-specific motifs. For consistency, we have also removed the TEXEL motif from our curated list of motifs since we wanted to focus on motifs that might mediate binding to host proteins. However, we added a reference to a study on TEXEL motifs (Coffee et al mBio 2018) as they provide a long list of TEXEL-containing proteins (lines 191-197).

7. The Discussion opens with speculative biological interpretation of the RON10-TRAF6 interaction and its potential role in NF-κB signaling and bradyzoite development. This framing presents the RON10 finding as the paper's headline result, when the primary contribution of the work is actually the curated motif dataset and prediction pipeline.
Good point, we have restructured the discussion section and now first discuss the perspectives and limitations of the resource prior to discussing the experimental results (lines 392-401).

Minor Comments
1. The statement "all these secreted proteins interact with host proteins throughout the infection cycle" (lines 113-115) is an overstatement and should be revised to reflect that only a subset of secreted proteins have demonstrated host interactions.
We have corrected this statement to specify that not all proteins from secretion organelles will interact with host proteins (line 114), as they might also just reside within the PV.

2. The claim that parasite protein export uses host machinery (lines 197-198) requires a specific citation supporting host machinery involvement in Toxoplasma effector translocation.
We have corrected this statement to differentiate that export of proteins is mediated by Toxoplasma's own processes and that further targeting to the host nucleus is mediated by host proteins (lines 185-199).

Reviewer #2 (Comments for the Author):

Many intracellular pathogens secrete effector proteins that interact with host proteins, often through short linear motifs on the pathogen proteins that bind to structured domains in host proteins. Identifying these motifs in secreted parasite proteins can provide valuable insight into their mechanistic functions. In this study, the authors created a computational discovery pipeline designed to mine databases for short linear motifs within the secretome of the apicomplexan parasite Toxoplasma gondii.

Identifying motifs in parasite proteins, particularly those with unknown functions, is a worthwhile approach that has previously led to important functional insights. For example, characterization of the GRA14 protein revealed TSG101 and ALIX-interacting motifs, which led to the discovery of its role in recruiting host ESCRT machinery to the parasitophorous vacuole (PV). In that case and others, the identification of motifs was performed on a single protein case. The pipeline employed in this study identified a number of potentially interesting motifs across the predicted secretome Toxoplasma.

However, the manuscript would benefit from a more detailed description of both the pipeline and the resulting analyses. The inclusion of in vitro binding assays to test predicted motifs is a strength. That said, these assays rely on short synthetic peptides (15 amino acids) interacting with a purified domain of TRAF6 known to bind the motif, instead of the full-length protein. This approach does not fully capture the biological context in which these interactions occur. A more informative experiment would be to use the peptides in pull-down assays with full-length TRAF6 from cellular lysates. Ideally, co-immunoprecipitation experiments would provide the most physiologically relevant validation of these predicted interactions.
We totally agree with the reviewer that our experimental results do not provide a full validation of the functional relevance and even existence of these protein interactions, and it would be very interesting to study those further. Unfortunately, the lab where most of the study has been conducted was mainly computational and has now closed due to the retirement of the PI, which is why it is extremely difficult to do more experimental work. We revised the manuscript in the results and discussion sections (lines 371, and 424-428) to describe the limitations and provide correct interpretations of our experimental results.

Specific comments:
Line 77: Including one or two examples of interactions between either virial or secreted bacterial effectors and host proteins, mediated by short linear motifs, would be informative.
We have included a couple of examples of how viral and bacterial effectors interact with host proteins via short linear motifs in the introduction (lines 73-80) that serve to illustrate their importance.

Lines 109-110: Dense granules are not membraneless organelles. They initially bud from the Golgi and are surrounded by a lipid bilayer. This description should be corrected.
We have corrected this statement (line 110) and added a recent reference to a review on their biogenesis.

Line 113: The statement, "All these proteins interact with host proteins throughout the infection cycle..." should be qualified. While this may be true for the parasite proteins that are inserted

into the PV membrane and are exported into the host cell, it may not necessarily be true of the parasite proteins confined to the PV lumen (line111). Please rephrase for accuracy.
We corrected this statement to specify that not all secreted proteins will interact with host proteins (line 114), as they might also just reside within the PV.

Line 135: Please include the URL for the Eukaryotic Linear Motif (ELM) reference.
We have included the URL of the ELM resource (line 135).

Lines 139-144: Please provide additional detail on motif classification and ELM classes. Specifically:
• Identify which two Toxoplasma motif instances were previously annotated (DOC), including motifs and corresponding protein.
• For the eleven novel motif instances, please list the motifs and proteins and explain the rationale for assigning them to the scaffolding class in ELM.
• Please list the eight additional novel motif instances and their associated proteins. Is there anything else known about these motifs? How were they identified if they could not be directly annotated using ELM?
We have revised the paragraph (lines 138-145) describing the curated motif instances and how they compare to ELM content. All of the motif instances we describe are also listed in Table 1. We have also added an extra column to Table 1 to annotate which motif instances were already in ELM, which ones were added to ELM thanks to our curation effort, and which ones cannot be added to ELM because the motif sequences do not match the regular expressions for any of the motif types defined in ELM. The latter ones we identified via literature searches, and not via computational sequence pattern searches in Toxoplasma proteins.

Lines 176-177: The description of GRA14 function should be expanded to note that recruitment of ESCRT (ALIX and TSG101) contributes not only to host cytosolic material uptake via PV membrane budding, but also to the internalization of host Rab vesicles.
We have updated the description of the GRA14 function to mention the internalisation of host Rab vesicles (182-183).

Table 1 (lines 179-181): Please define the ELM motif abbreviations used in the table.
We are now defining the ELM motif abbreviations in the legend of Table 1 (lines 153-155) as well as in the results text (lines 138-145).

Line 190: Clarify what is meant by "during establishment".
We have changed the sentence to the following: “Further during colonisation and formation of an intracellular niche, more parasite proteins are secreted into the host cytosol by the parasite machinery and subsequently may be transported to the host nucleus by the host cellular machinery.” (lines 185-187).

Line 193-194: Specify the motif from GRA24 that binds to MAPK14
We have added the name of the motif as kinase-interacting motifs (KIM) to the results section (lines 138-140) and Table 1.

Lines 196-198: The description of protein export is unclear and appears to conflate distinct processes. Please distinguish between the export of parasite proteins from the PV, which

relies on parasite machinery, and the trafficking of parasite proteins within the host cell, such as to the nucleus, which uses host machinery.

We have revised this paragraph accordingly and now specify that export requires parasite machinery and the trafficking of parasite proteins within the host cell requires host machinery. We use GRA16 as an example for a protein that is transported by both processes, once involving the TEXEL motif and once its NLS (lines 191-199).

Lines 222-224: A clearer and more detailed description of the computational pipeline is needed. Clarify whether this refers to a novel algorithm or a workflow built on existing database search tools. For the ELM database searches using "regular expressions defined for annotated motif classes" please specify which expressions were used.

We have revised this entire paragraph to better explain the different prediction, annotation and filtering steps and which ones of those are conceptually based on previous motif prediction pipelines and which ones are specific to this pipeline (lines 208-246). We have also added details about the use of regular expressions to predict motif occurrences. We also added to the discussion a small part on the existing resource ToxoDB that offers a simple regular expression search for proteomes, but the output is for a single regular expression, while our pipeline produces motif predictions for 297 motif types (lines 396-401).

Re: Spectrum00724-26R1 (**Short linear motifs - Underexplored players driving Toxoplasma gondii infection**)

Dear Dr. Jesus Alvarado Valverde:

Your manuscript has been accepted, and I am forwarding it to the ASM production staff for publication. Your paper will first be checked to make sure all elements meet the technical requirements. ASM staff will contact you if anything needs to be revised before copyediting and production can begin. Otherwise, you will be notified when your proofs are ready to be viewed.

Data Availability: ASM policy requires that data be available to the public upon online posting of the article, so please verify all links to sequence records, if present, and make sure that each number retrieves the full record of the data. If a new accession number is not linked or a link is broken, provide production staff with the correct URL for the record. If the accession numbers for new data are not publicly accessible before the expected online posting of the article, publication may be delayed; please contact ASM production staff immediately with the expected release date.

Publication Fees: For information on publication fees and which article types have charges, please visit ourwebsite. We have partnered with Copyright Clearance Center (CCC) to collect author charges. If fees apply to your paper, you will receive a message from no-reply@copyright.com with further instructions. For questions related to paying charges through RightsLink, please contact CCC at ASM_Support@copyright.com or toll free at +1-877-622-5543. CCC makes every attempt to respond to all emails within 24 hours.

ASM Membership: Corresponding authors may join or renew ASM membership to obtain discounts on publication fees. Need to upgrade your membership level? Please contact Customer Service at Service@asmusa.org.

Embargo Policy: A press release may be issued as soon as the manuscript is posted on theSpectrum Latest Articles webpage. The corresponding author will receive an email with the subject line "ASM Journals Author Services Notification" when the article is available online.

The ASM Journals program strives for constant improvement in our submission and publication process. Please tell us how we can improve your experience by taking this quick Author Survey.

Thank you for submitting your paper to Spectrum.

Sincerely,
Prakash Srinivasan
Editor
Microbiology Spectrum
