# Supplementary material for: Short linear motifs—underexplored players driving Toxoplasma gondii infection
Source: Microbiol Spectr. 2026 Aug 5;14(9):e00724-26. doi: 10.1128/spectrum.00724-26 (PMC13532356; doi:10.1128/spectrum.00724-26)
Supplement: Supplementary file 2 [file spectrum.00724-26-s0002.pdf]

Supplementary Data - MST results

TgRON10 (peptide ID: 10784)
Sequence: 5-Fluo-TNGTPE**E**YEET-Amid

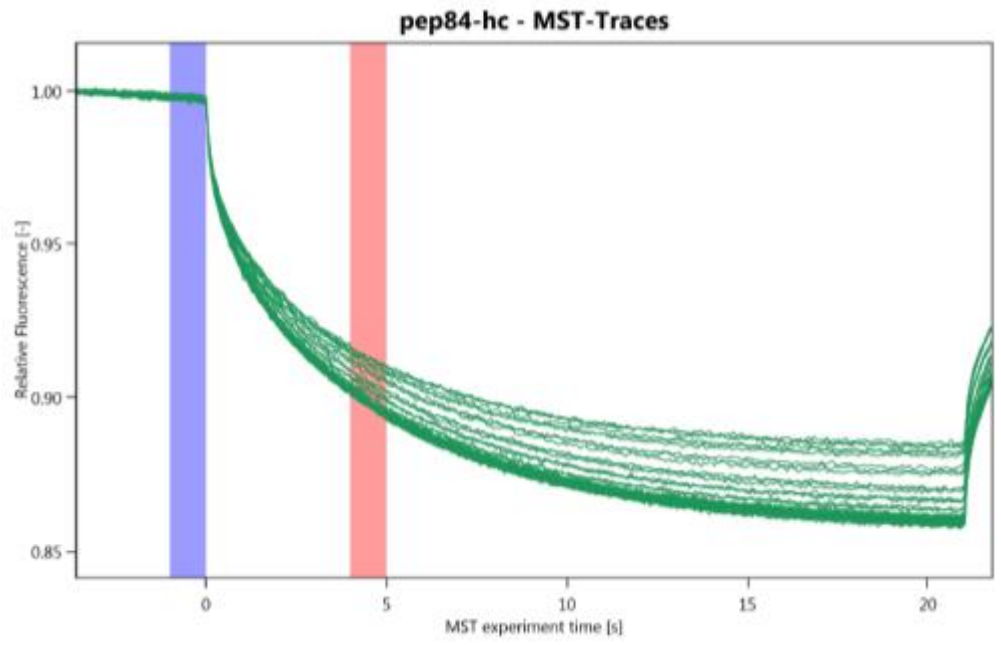

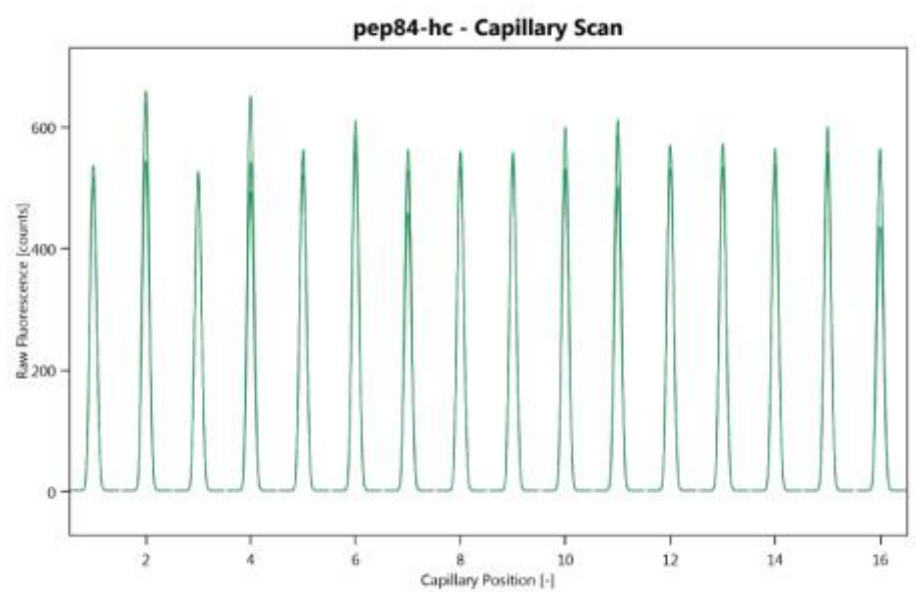

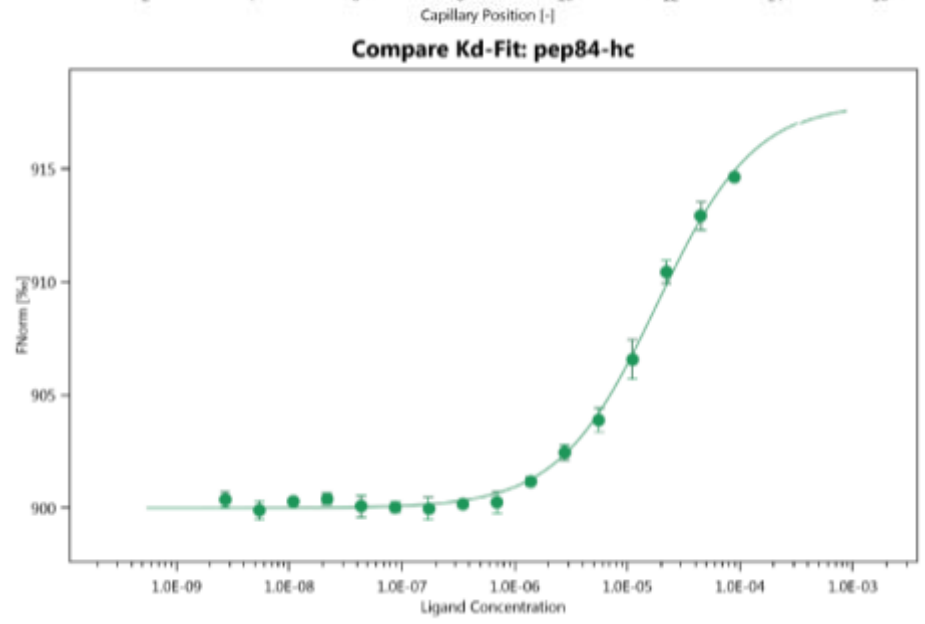

Dataset Overview

Name:	hTRAF6 Tray
Graph Color:	•
Target Name:	pep84
Target Concentration:	50 nM
Ligand Name:	hTRAF6
Ligand Concentration:	8.91E+04 nM to 2.72 nM
n:	3
Comments:	
Excitation Power:	20%
MST Power:	40%
Temperature:	25.0°C
Kd:	1.8214E-05
Kd Confidence:	± 1.4653E-06
Response Amplitude:	17.985219
TargetConc:	5E-08[Fixed]
Unbound:	899.98
Bound:	917.97
Std. Error of Regression:	0.3041618
Reduced χ^2:	2.0670054
Signal to Noise:	63.516276

Conclusion: binds (Kd = 18.2µM)

TgRON10 mutant (peptide ID: 10785)
Sequence: 5-Fluo-TNGTPE**S**YEET-Amid

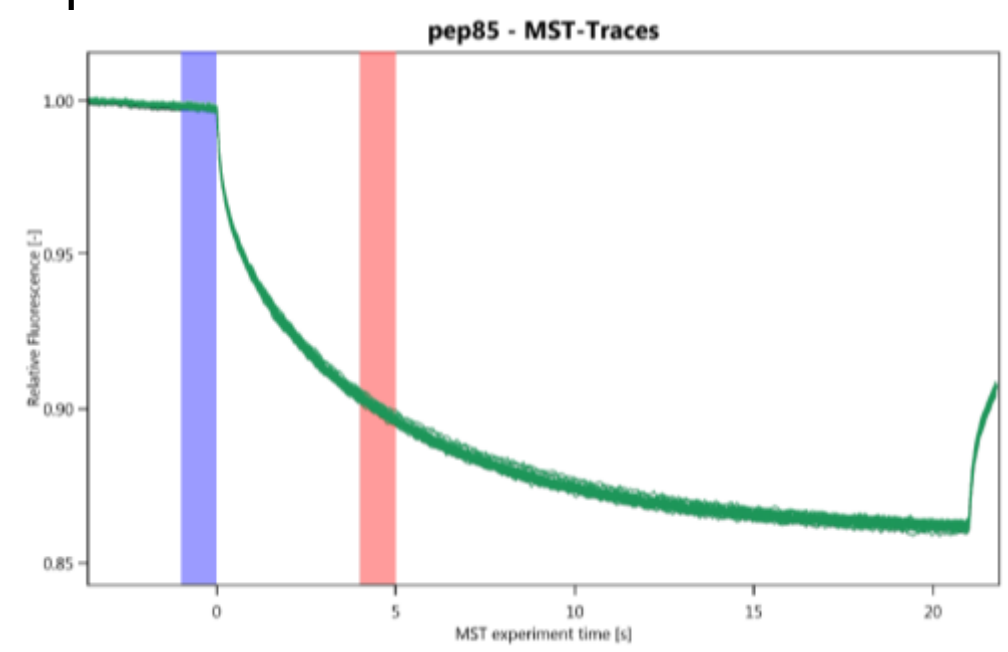

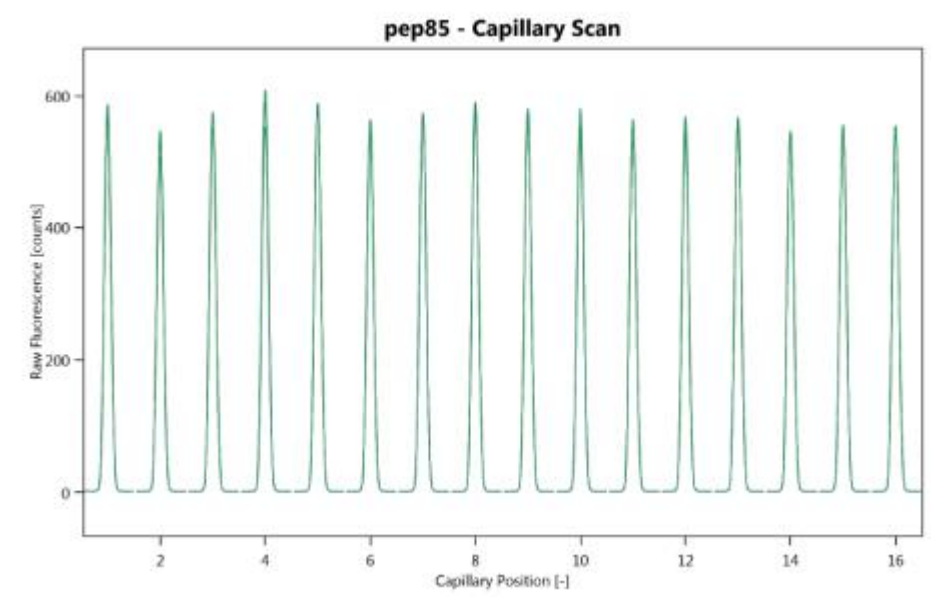

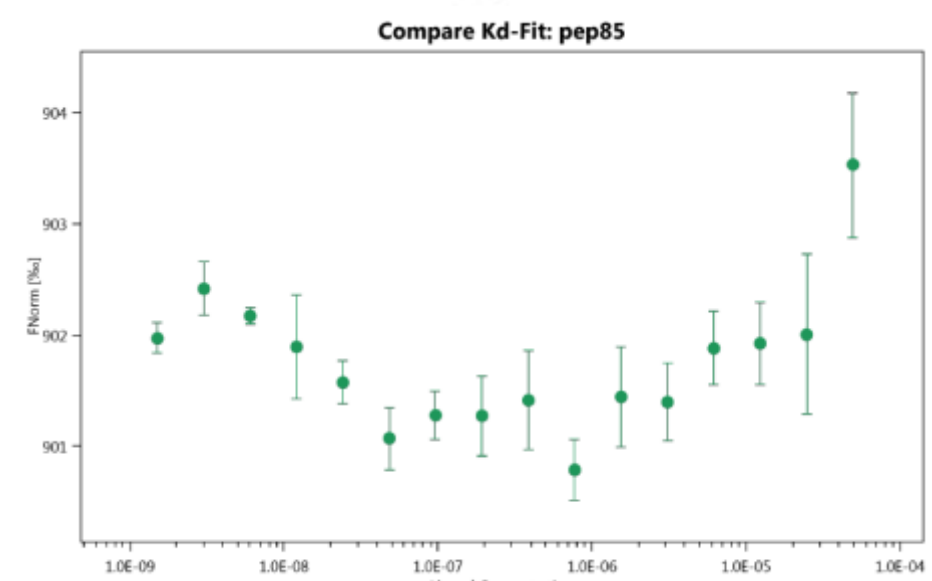

Dataset Overview

Name:	hTRAF6 Tray
Graph Color:	•
Target Name:	pep85
Target Concentration:	50 nM
Ligand Name:	hTRAF6
Ligand Concentration:	49.5 µM to 0.00151 µM
n:	3
Comments:	
Excitation Power:	20%
MST Power:	40%
Temperature:	25.0°C
Kd:	
Kd Confidence:	
Response Amplitude:	
TargetConc:	5E-08[Fixed]
Unbound:	
Bound:	
Std. Error of Regression:	
Reduced χ^2:	
Signal to Noise:	

Conclusion: no binding

TgRON6 (peptide ID: 10786)
Sequence: 5-Fluo-HERAPG**E**GGHE-Amid

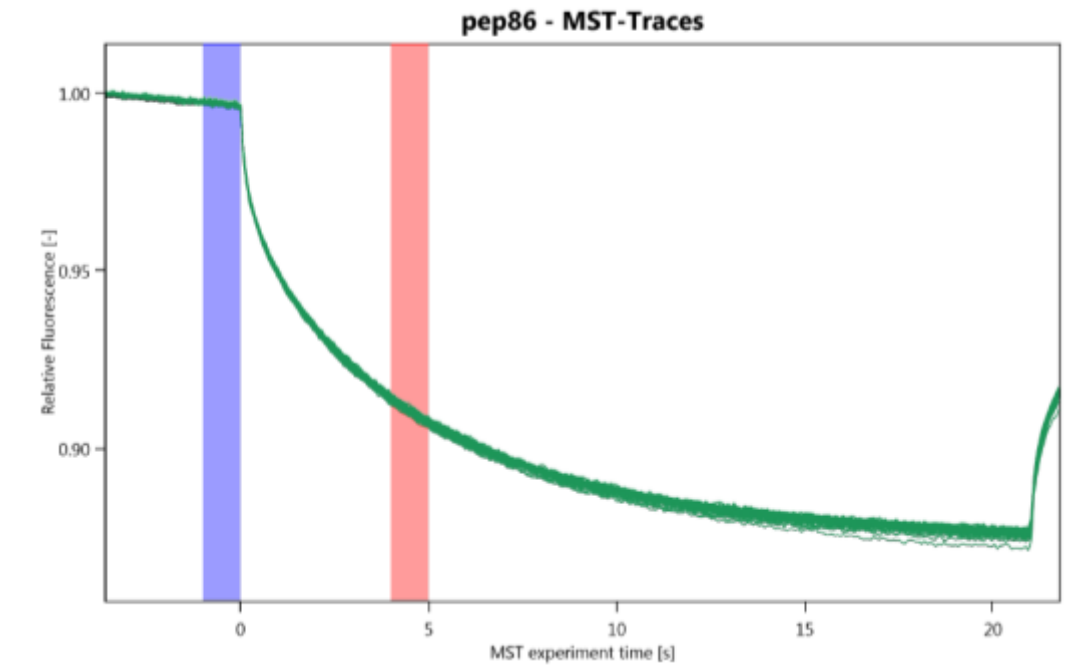

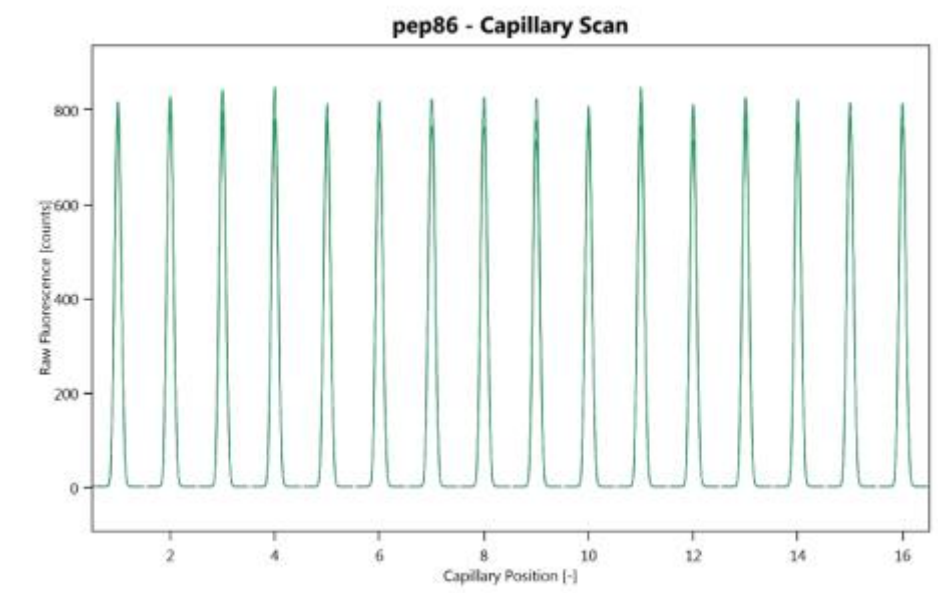

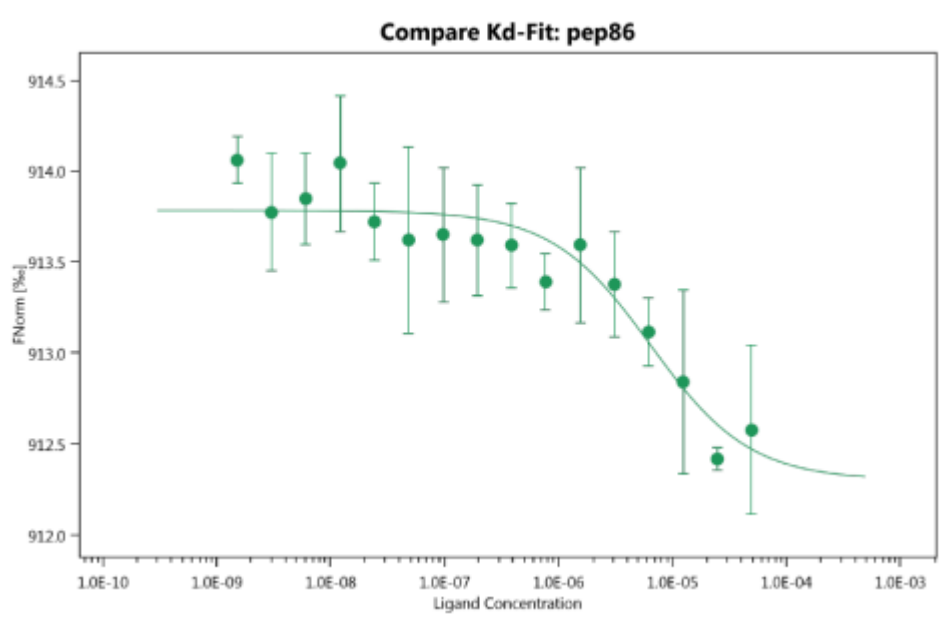

Dataset Overview

Name:	hTRAF6 Tray
Graph Color:	•
Target Name:	pep86
Target Concentration:	50 nM
Ligand Name:	hTRAF6
Ligand Concentration:	49.5 µM to 0.00151 µM
n:	3
Comments:	
Excitation Power:	20%
MST Power:	40%
Temperature:	25.0°C
Kd:	6.4052E-06
Kd Confidence:	± 2.854E-06
Response Amplitude:	1.479834
TargetConc:	5E-08[Fixed]
Unbound:	913.78
Bound:	912.3
Std. Error of Regression:	0.16039139
Reduced χ^2:	1.3669731
Signal to Noise:	9.9107359

Conclusion: no binding

TgRON6 mutant (peptide ID: 10787)
Sequence: 5-Fluo-HERAPG**S**GGHE-Amid

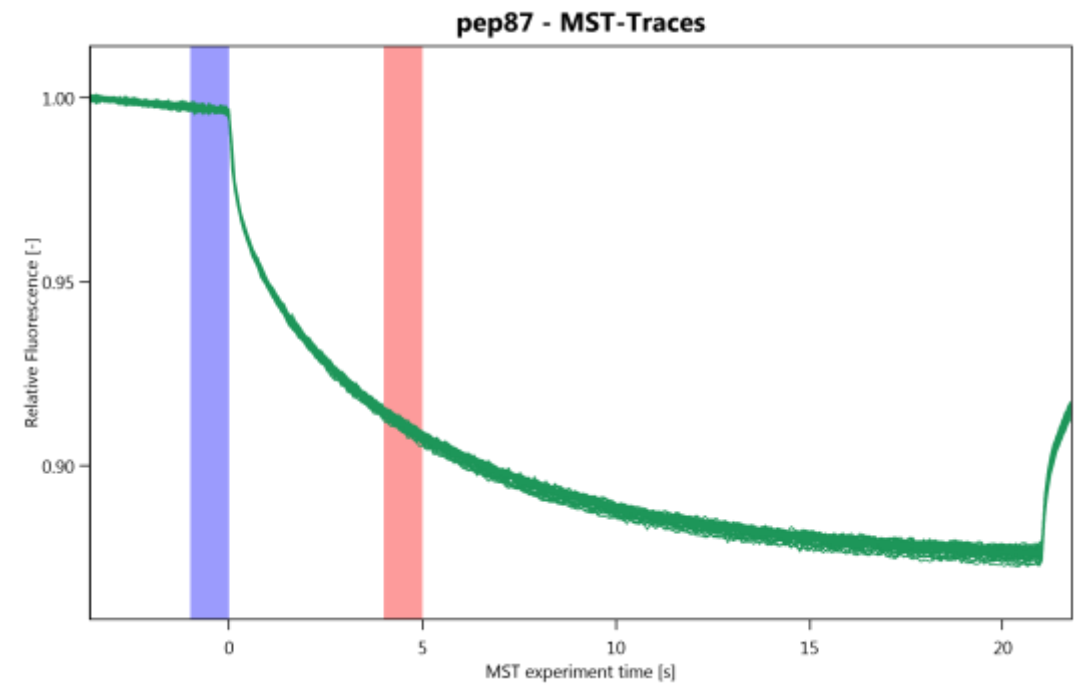

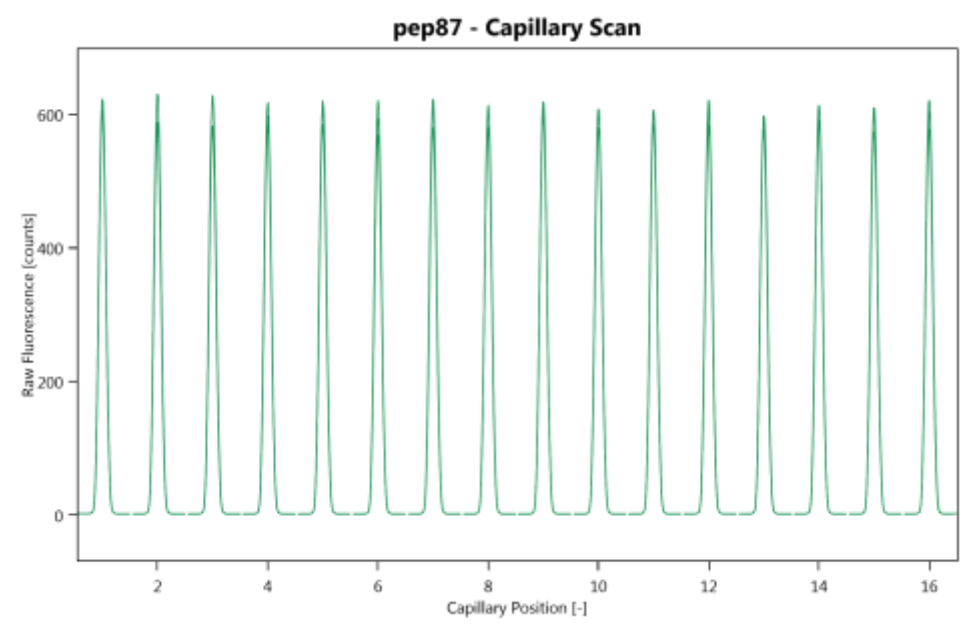

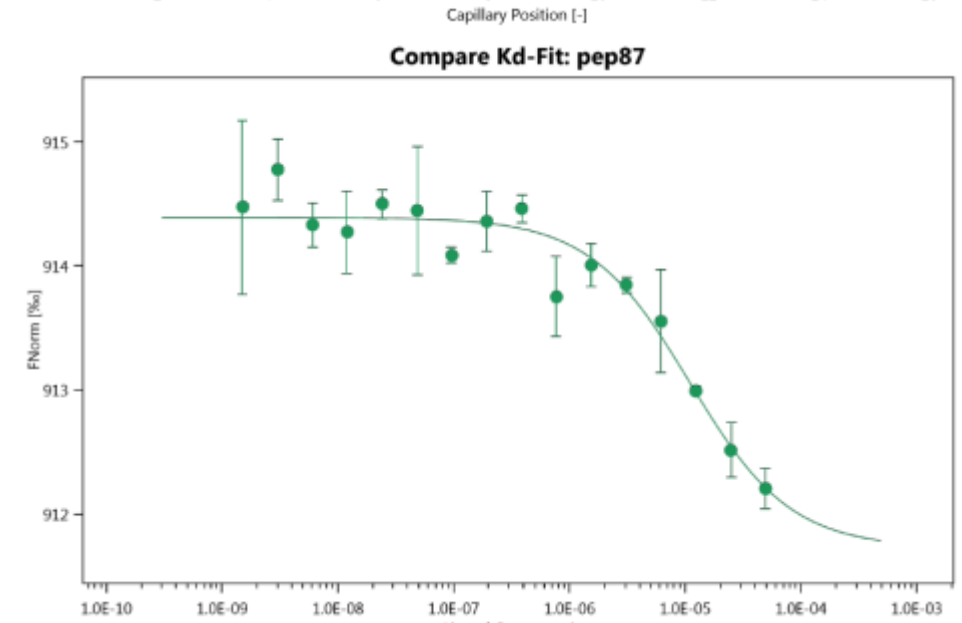

Dataset Overview

Name:	hTRAF6 Tray
Graph Color:	•
Target Name:	pep87
Target Concentration:	50 nM
Ligand Name:	hTRAF6
Ligand Concentration:	49.5 µM to 0.00151 µM
n:	3
Comments:	
Excitation Power:	20%
MST Power:	40%
Temperature:	25.0°C
Kd:	1.0955E-05
Kd Confidence:	± 4.1052E-06
Response Amplitude:	2.6642171
TargetConc:	5E-08[Fixed]
Unbound:	914.39
Bound:	911.72
Std. Error of Regression:	0.20315397
Reduced χ^2:	2.1338393
Signal to Noise:	14.086992

Conclusion: no binding

TgGRA7 (peptide ID: 20443)
Sequence: 5-Fluo-LETEPD**E**QEEV-Amid

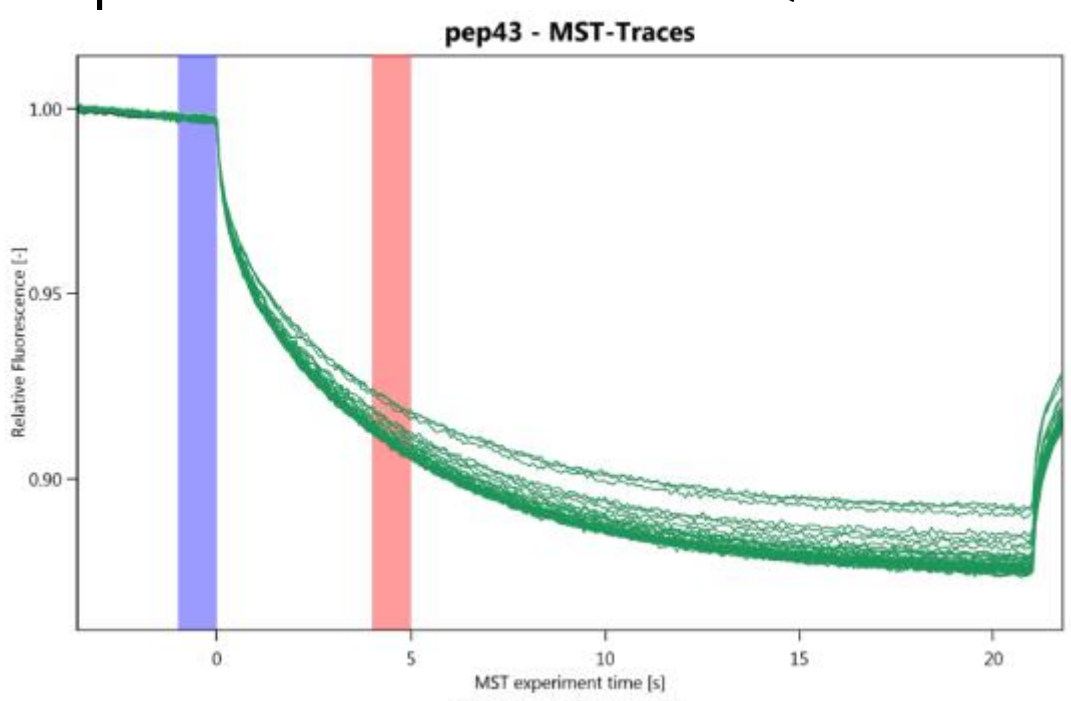

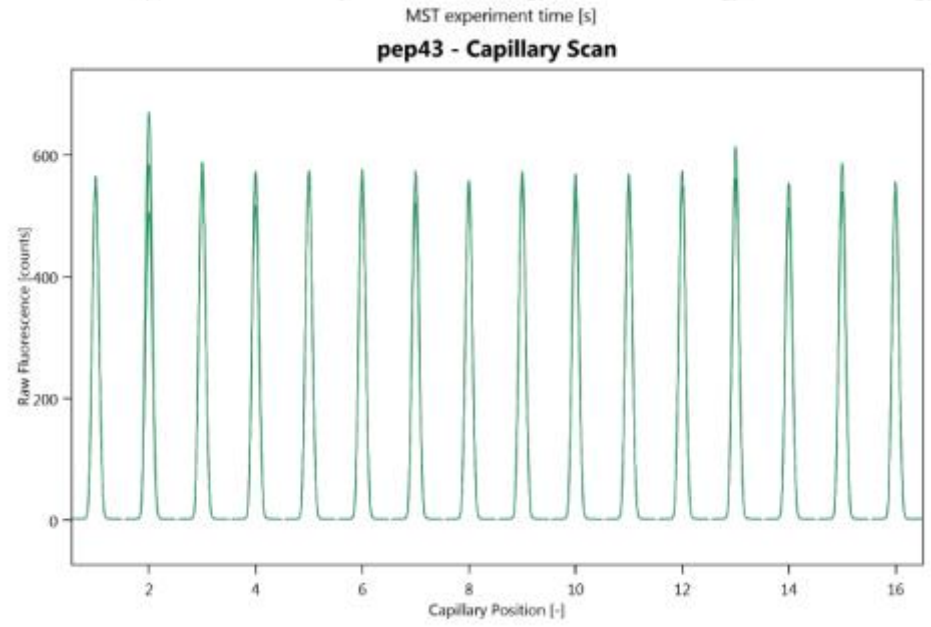

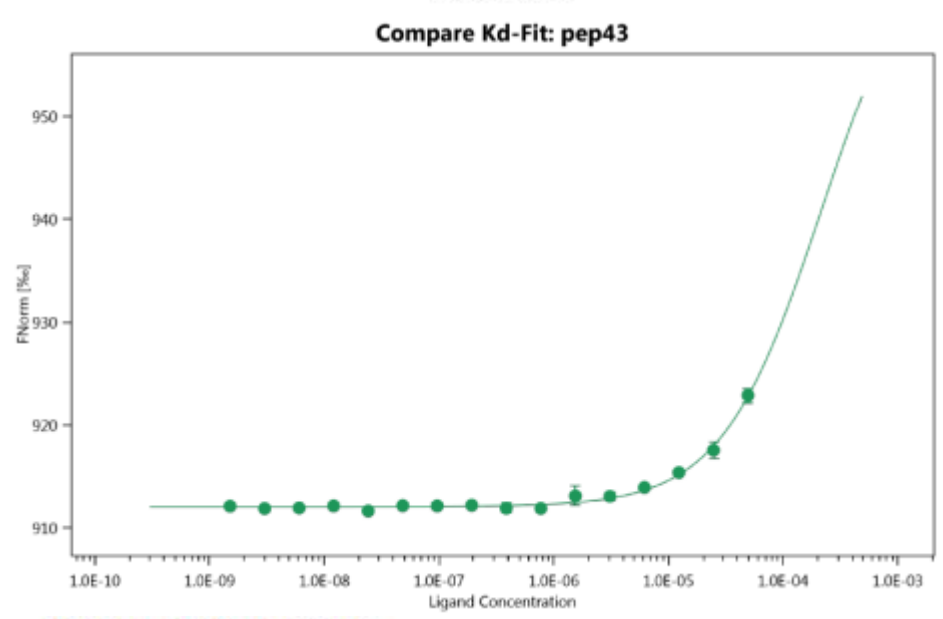

Dataset Overview

Name:	hTRAF6 Tray
Graph Color:	•
Target Name:	pep43
Target Concentration:	50 nM
Ligand Name:	hTRAF6
Ligand Concentration:	49.5 µM to 0.00151 µM
n:	3
Comments:	
Excitation Power:	20%
MST Power:	40%
Temperature:	25.0°C
Kd:	0.00021695
Kd Confidence:	± 0.0001174
Response Amplitude:	57.732424
TargetConc:	5E-08[Fixed]
Unbound:	912.05
Bound:	969.78
Std. Error of Regression:	0.30432054
Reduced χ^2:	0.6919831
Signal to Noise:	203.78043

Conclusion: very weak binding

TgGRA7 mutant (peptide ID: 20444)
Sequence: 5-Fluo-LETEPD**S**QEEV-Amid

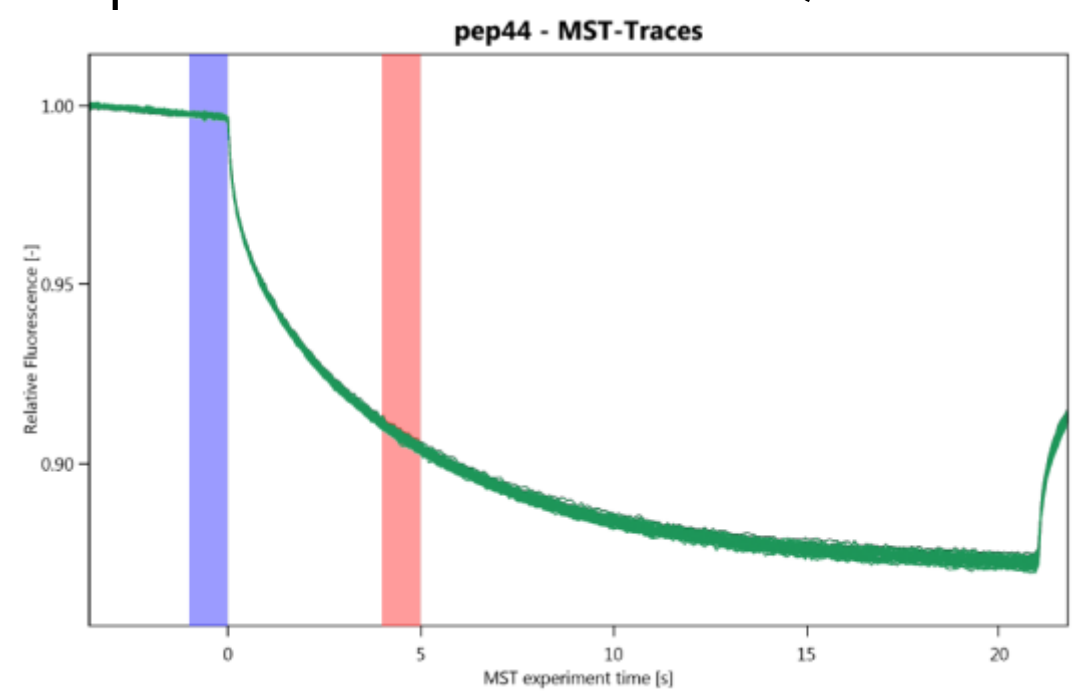

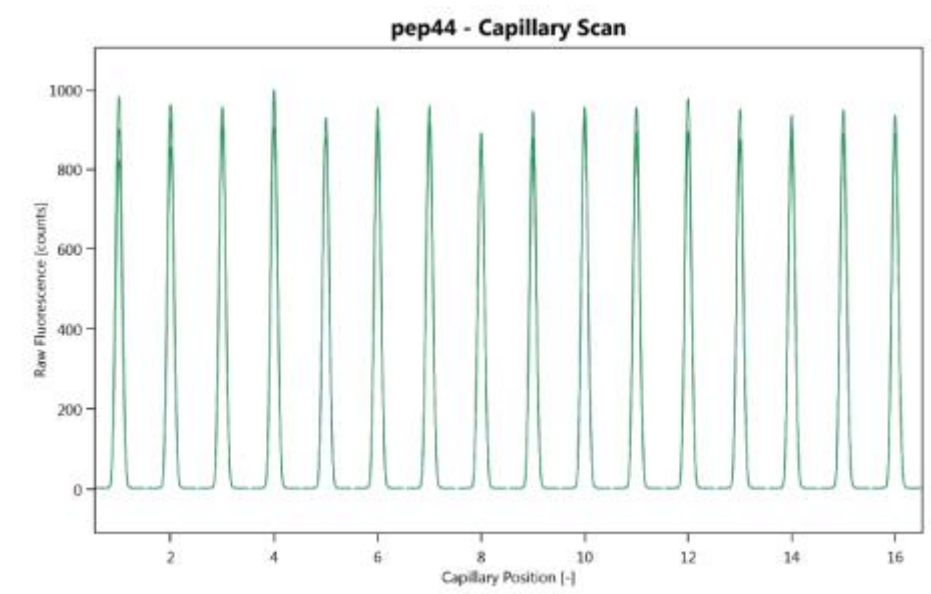

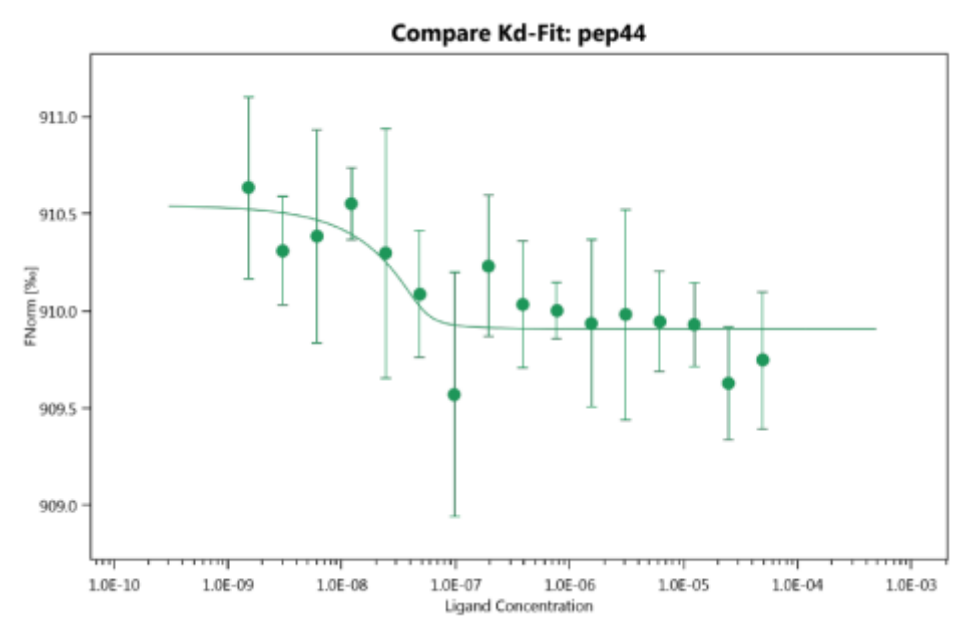

Dataset Overview

Name:	hTRAF6 Tray
Graph Color:	•
Target Name:	pep44
Target Concentration:	50 nM
Ligand Name:	hTRAF6
Ligand Concentration:	49.5 µM to 0.00151 µM
n:	3
Comments:	
Excitation Power:	20%
MST Power:	40%
Temperature:	25.0°C
Kd:	1.6596E-09
Kd Confidence:	± 6.4862E-09
Response Amplitude:	0.63489257
TargetConc:	5E-08[Fixed]
Unbound:	910.54
Bound:	909.91
Std. Error of Regression:	0.18643298
Reduced χ^2:	0.31914315
Signal to Noise:	3.6580654

Conclusion: no binding

TgGRA15 (peptide ID: 20445)
Sequence: 5-Fluo-QPQVPG**E**NSYY-Amid

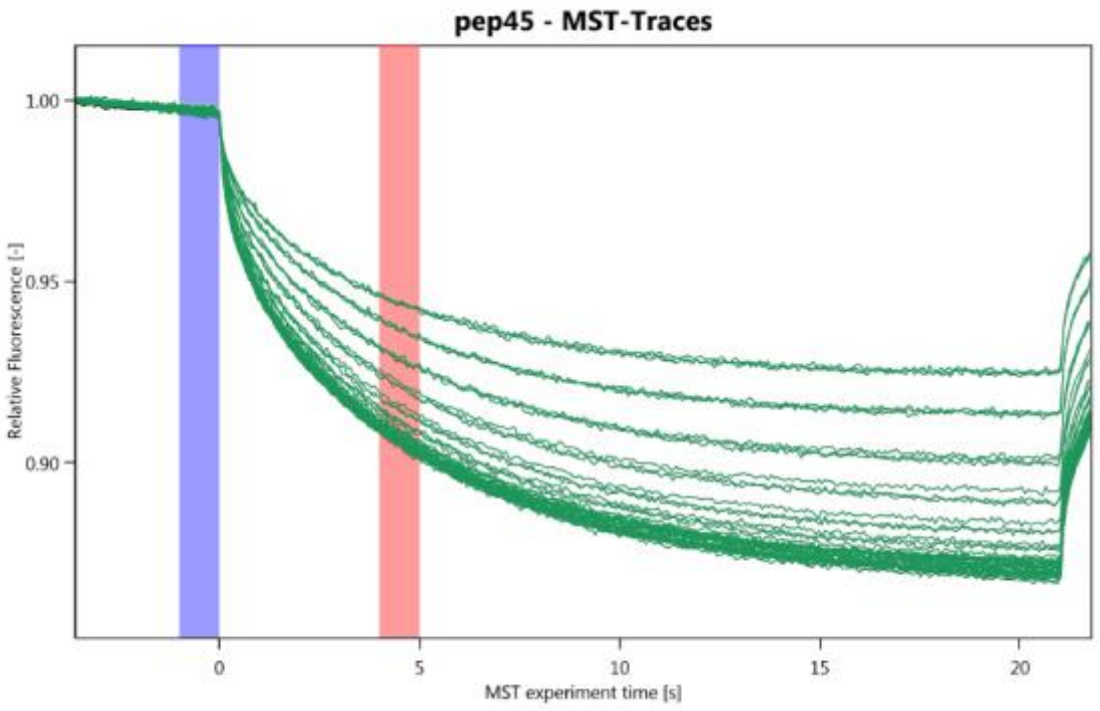

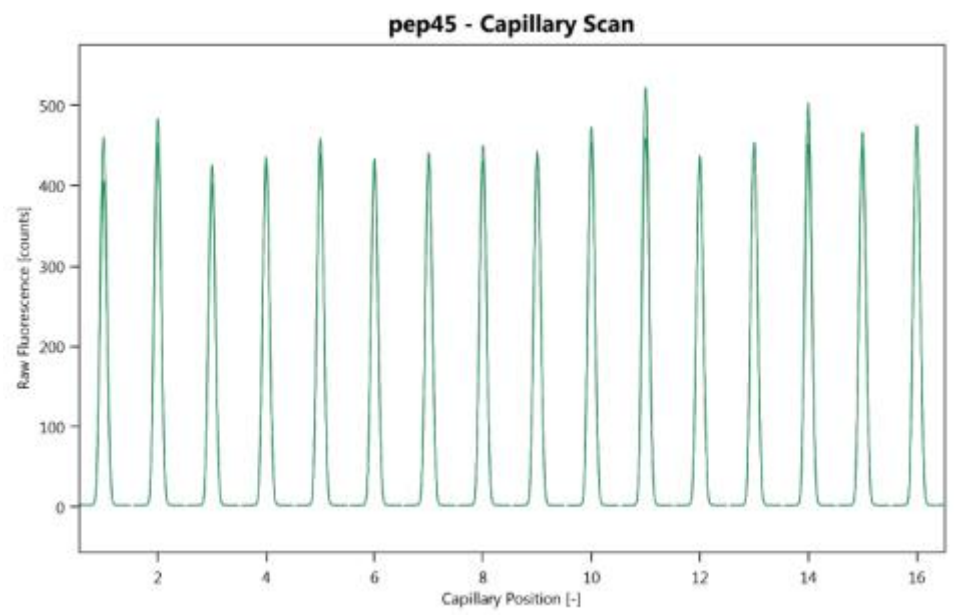

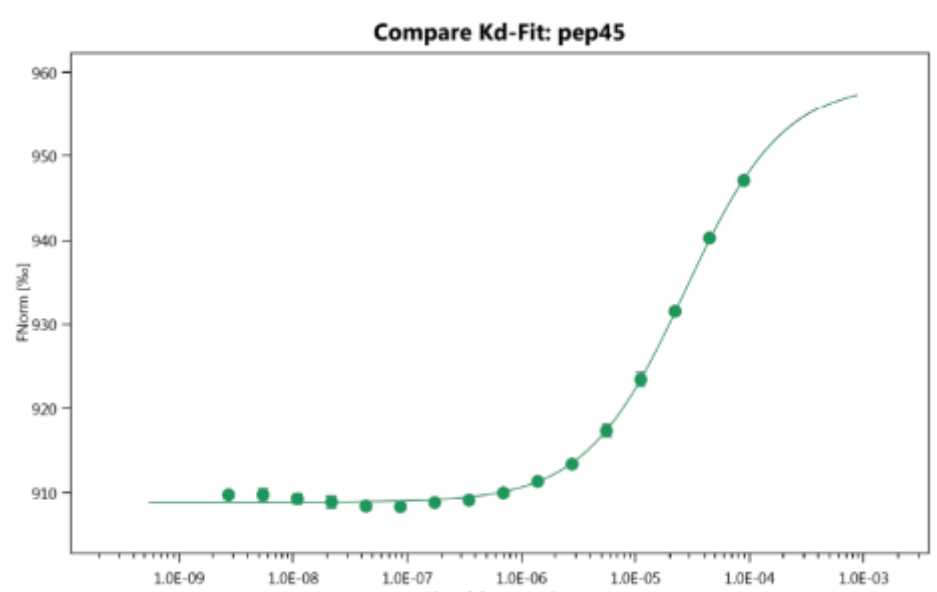

Dataset Overview

Name:	hTRAF6 Tray
Graph Color:	•
Target Name:	pep45
Target Concentration:	50 nM
Ligand Name:	hTRAF6
Ligand Concentration:	8.91E+04 nM to 2.72 nM
n:	3
Comments:	
Excitation Power:	20%
MST Power:	40%
Temperature:	25.0°C
Kd:	2.6913E-05
Kd Confidence:	± 1.4303E-06
Response Amplitude:	49.948887
TargetConc:	5E-08[Fixed]
Unbound:	908.86
Bound:	958.81
Std. Error of Regression:	0.47059985
Reduced χ^2:	1.1021555
Signal to Noise:	114.01132

Conclusion: binds (Kd = 26.9μM)

TgGRA15 mutant (peptide ID: 20446)
Sequence: 5-Fluo-QPQVPG**S**NSYY-Amid

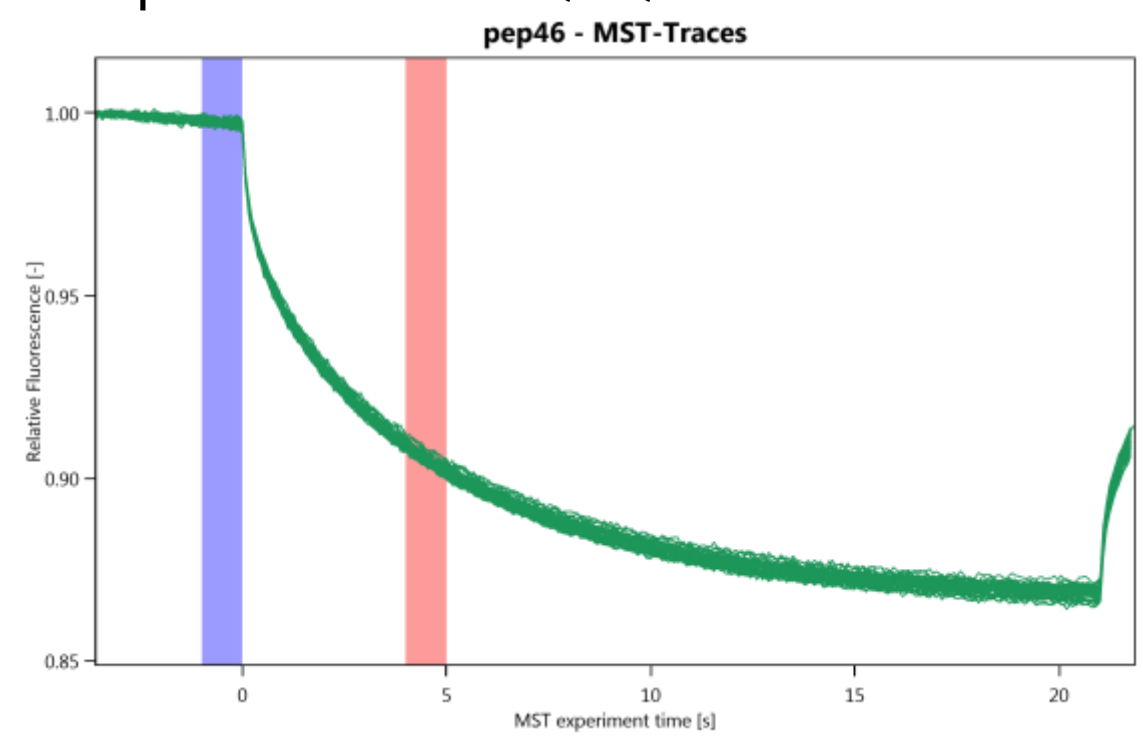

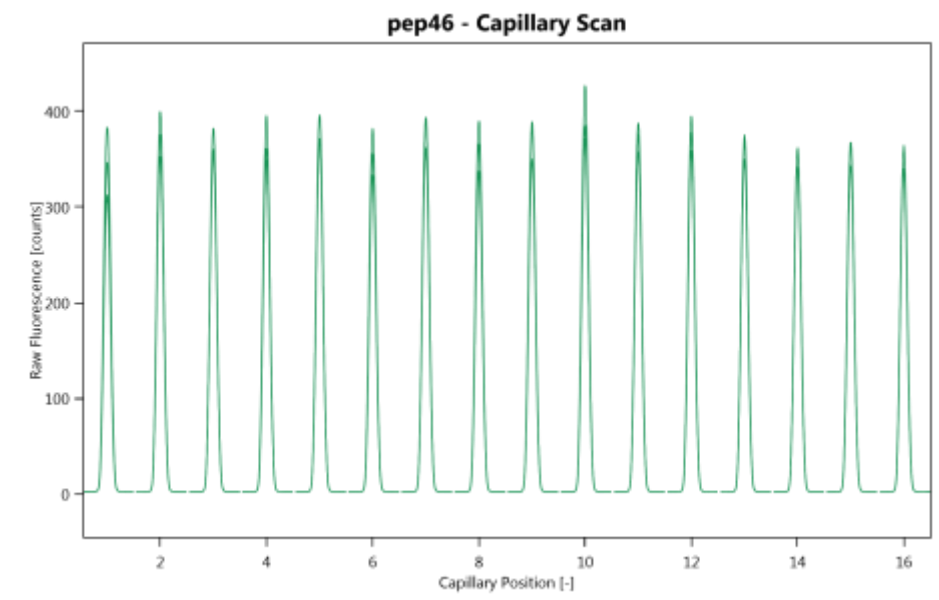

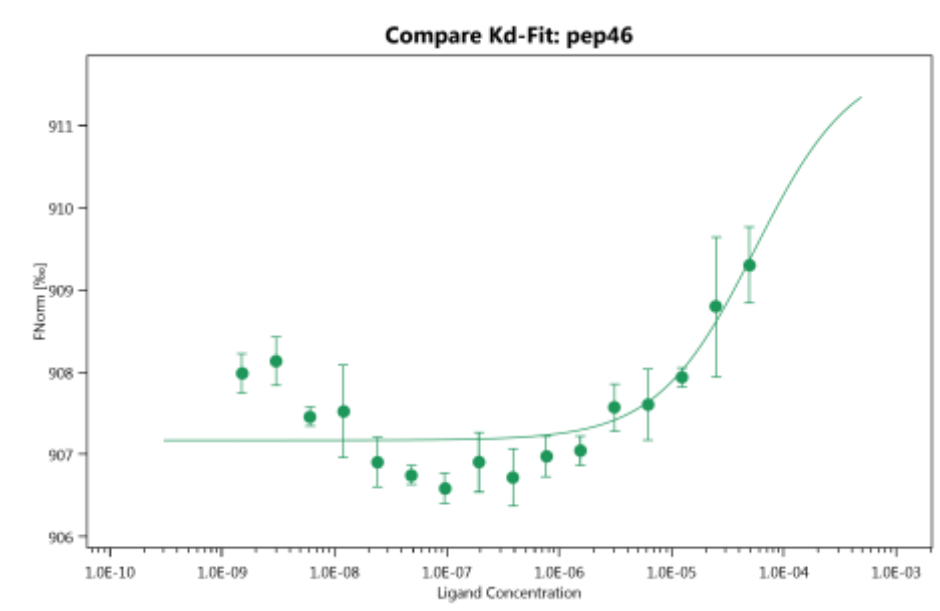

Dataset Overview

Name:	hTRAF6 Tray
Graph Color:	•
Target Name:	pep46
Target Concentration:	50 nM
Ligand Name:	hTRAF6
Ligand Concentration:	49.5 μM to 0.00151 μM
n:	3
Comments:	
Excitation Power:	20%
MST Power:	40%
Temperature:	25.0°C
Kd:	5.5147E-05
Kd Confidence:	± 8.6559E-05
Response Amplitude:	4.6468734
TargetConc:	5E-08[Fixed]
Unbound:	907.17
Bound:	911.82
Std. Error of Regression:	0.47406161
Reduced χ^2:	4.606408
Signal to Noise:	10.529312

Conclusion: no binding

Human MAVS (peptide ID: 10788)
Sequence: 5-Fluo-PSHGPE**E**NEYK-Amid

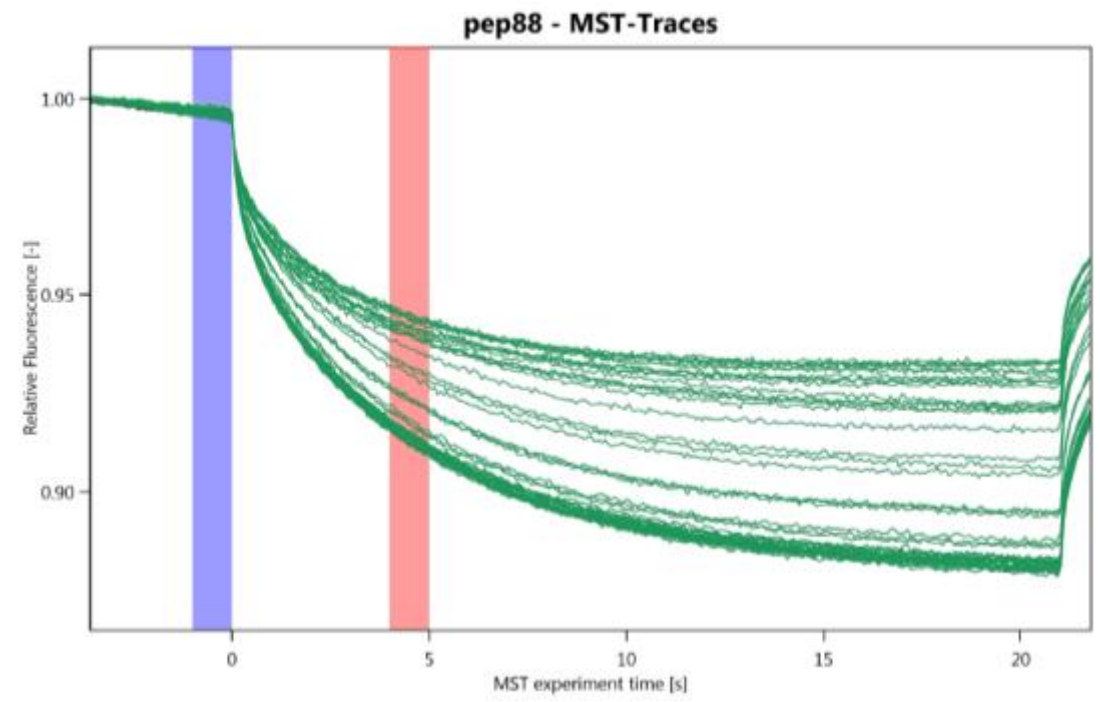

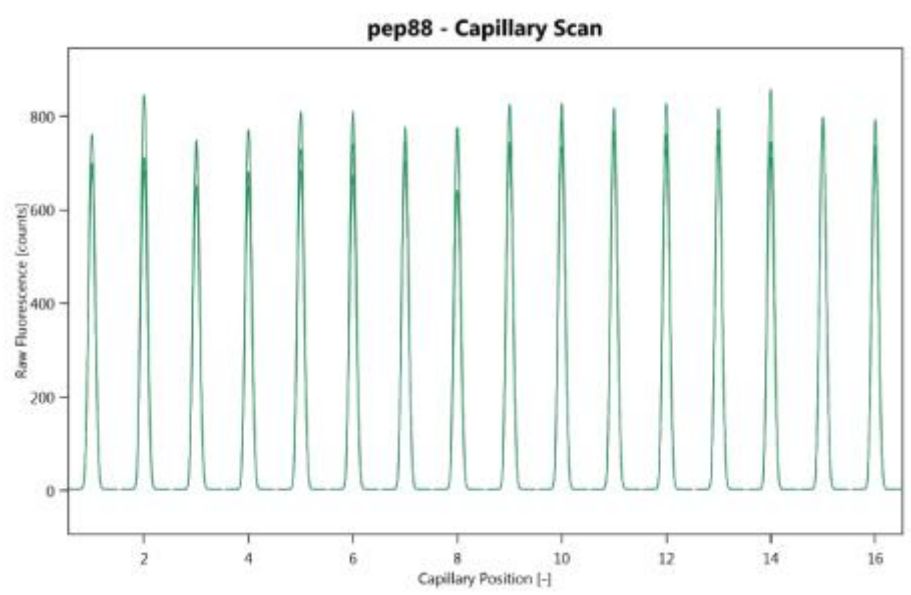

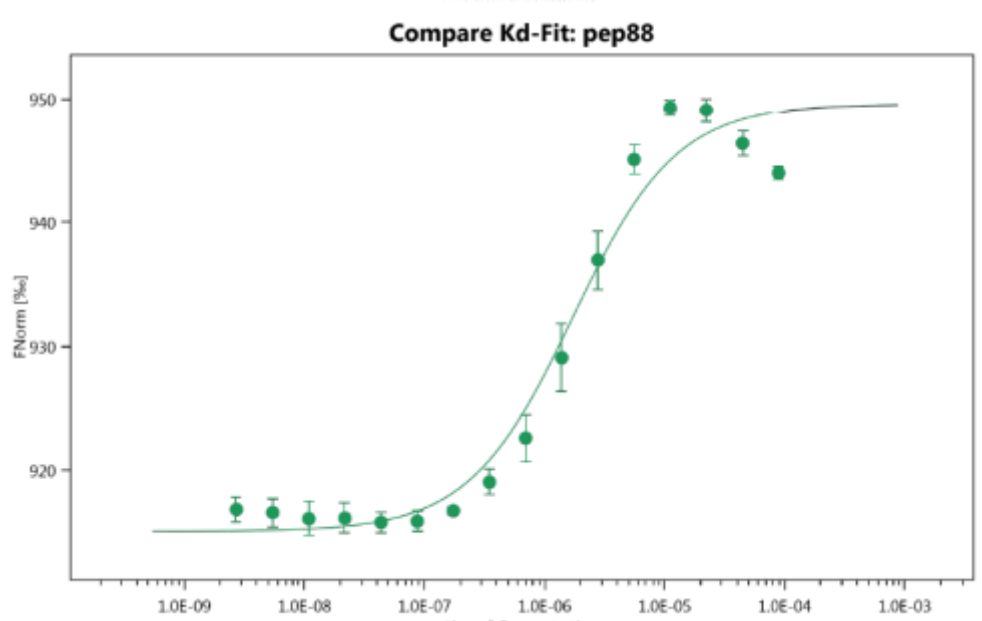

Dataset Overview

Name:	hTRAF6 Tray
Graph Color:	•
Target Name:	pep88
Target Concentration:	50 nM
Ligand Name:	hTRAF6
Ligand Concentration:	8.91E+04 nM to 2.72 nM
n:	3
Comments:	
Excitation Power:	20%
MST Power:	40%
Temperature:	25.0°C
Kd:	1.6913E-06
Kd Confidence:	± 3.8719E-07
Response Amplitude:	34.532069
TargetConc:	5E-08[Fixed]
Unbound:	915.06
Bound:	949.59
Std. Error of Regression:	2.5299539
Reduced χ^2:	12.937886
Signal to Noise:	14.661687

Conclusion: binds (Kd = 1.69μM)

Human MAVS mutant (peptide ID: 10789)
Sequence: 5-Fluo-PSHGPE**S**NEYK-Amid

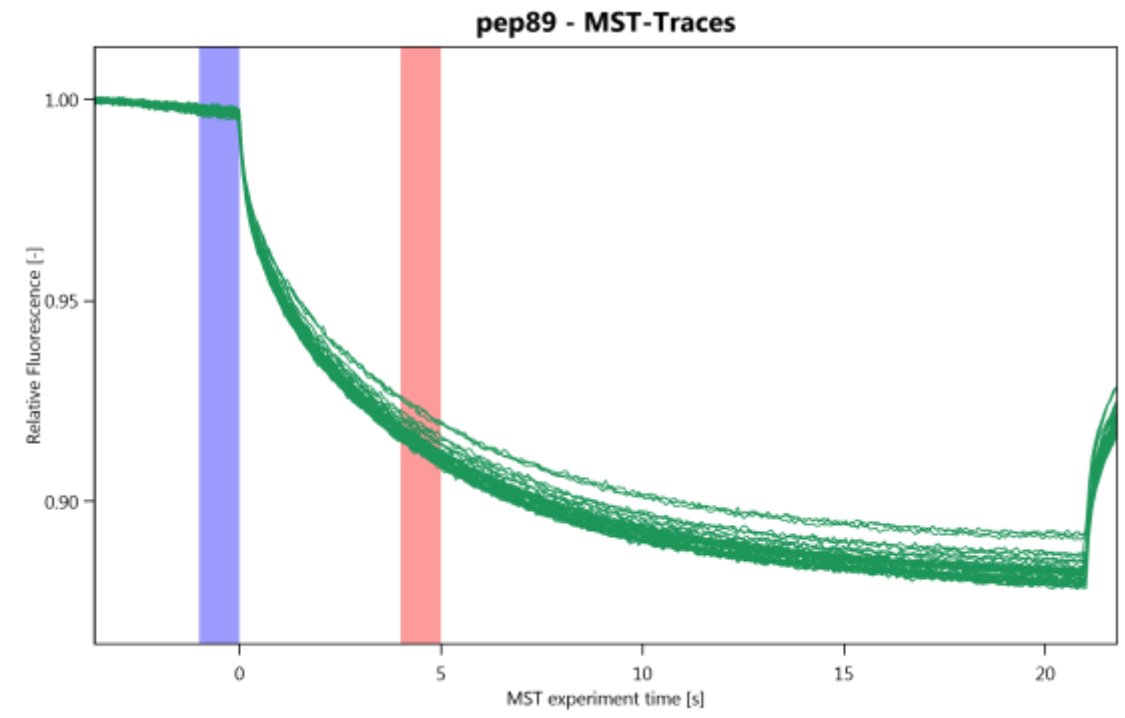

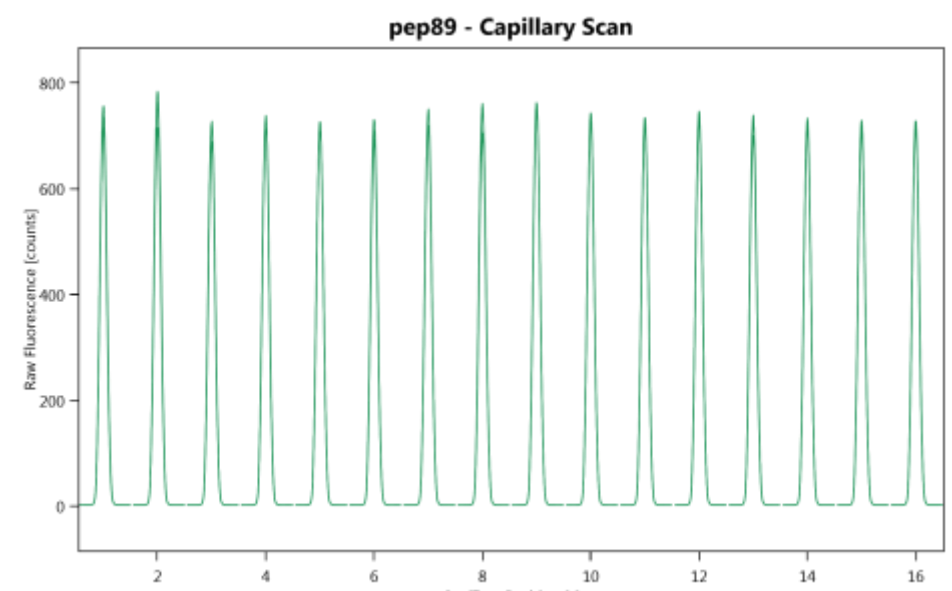

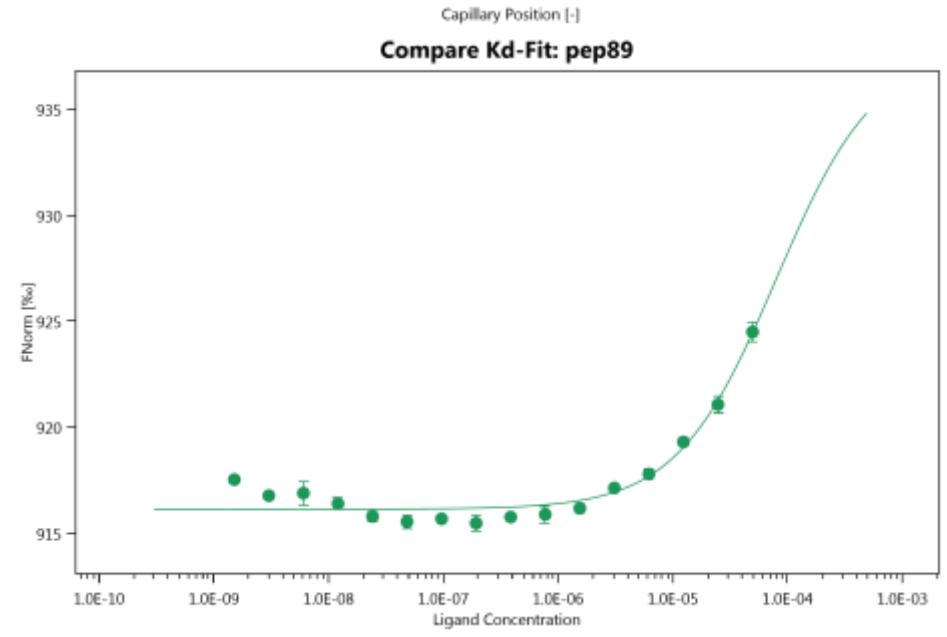

Dataset Overview

Name:	hTRAF6 Tray
Graph Color:	•
Target Name:	pep89
Target Concentration:	50 nM
Ligand Name:	hTRAF6
Ligand Concentration:	49.5 μM to 0.00151 μM
n:	3
Comments:	
Excitation Power:	20%
MST Power:	40%
Temperature:	25.0°C
Kd:	7.9795E-05
Kd Confidence:	± 5.3616E-05
Response Amplitude:	21.742605
TargetConc:	5E-08[Fixed]
Unbound:	916.14
Bound:	937.88
Std. Error of Regression:	0.61703124
Reduced χ^2:	16.350615
Signal to Noise:	37.851089

Conclusion: weak binding
