# Supplementary material for: Short linear motifs—underexplored players driving Toxoplasma gondii infection
Source: Microbiol Spectr. 2026 Aug 5;14(9):e00724-26. doi: 10.1128/spectrum.00724-26 (PMC13532356; doi:10.1128/spectrum.00724-26)
Supplement: Supplementary file 3 [file spectrum.00724-26-s0001.docx]

**Supplementary information**

**Supplementary Figure 1 - Protein disorder content in different subcellular compartments and summary statistics of motif matches per motif class. A**. IUPred3 mean score for proteins of different Toxoplasma subcellular locations as determined from hyperLOPIT. **B**. Counts of motif matches of the most frequent motif types per motif class.

**Table S1** – Motif predictions in microneme, rhoptry and dense granule proteins

**Table S2** – Motif predictions in 42 experimentally tested secreted rhoptry and dense granule proteins

**Table S3** – Selected TRAF6 motif-containing peptides and binding assay results

**Data S1** – MST binding extended results for the selected TRAF6 motif-containing peptides
